# Data Driven Mathematical Model of Colon Cancer Progression

**DOI:** 10.3390/jcm9123947

**Published:** 2020-12-05

**Authors:** Arkadz Kirshtein, Shaya Akbarinejad, Wenrui Hao, Trang Le, Sumeyye Su, Rachel A. Aronow, Leili Shahriyari

**Affiliations:** 1 Department of Mathematics and Statistics, University of Massachusetts Amherst, Amherst, MA 01003-9305, USA; akirshtein@umass.edu (A.K.); sakbarinejad@umass.edu (S.A.); tramle@umass.edu (T.L.); sumeyyesu@umass.edu (S.S.); aronow@math.umass.edu (R.A.A.); 2 Department of Mathematics, Pennsylvania State University, University Park, State College, PA 16802, USA; wxh64@psu.edu

**Keywords:** colon cancer, data driven mathematical model, immune pattern, sensitivity analysis, gene expression profiles, tumor deconvolution, macrophages, T-cells, dendritic cells, HMGB1

## Abstract

Every colon cancer has its own unique characteristics, and therefore may respond differently to identical treatments. Here, we develop a data driven mathematical model for the interaction network of key components of immune microenvironment in colon cancer. We estimate the relative abundance of each immune cell from gene expression profiles of tumors, and group patients based on their immune patterns. Then we compare the tumor sensitivity and progression in each of these groups of patients, and observe differences in the patterns of tumor growth between the groups. For instance, in tumors with a smaller density of naive macrophages than activated macrophages, a higher activation rate of macrophages leads to an increase in cancer cell density, demonstrating a negative effect of macrophages. Other tumors however, exhibit an opposite trend, showing a positive effect of macrophages in controlling tumor size. Although the results indicate that for all patients the size of the tumor is sensitive to the parameters related to macrophages, such as their activation and death rate, this research demonstrates that no single biomarker could predict the dynamics of tumors.

## 1. Introduction

Recent studies show that many cancers arise from sites of chronic inflammation [1,2,3,4]. Balkwill et al. [5] provide a list of inflammatory conditions that predispose an individual to cancer, in particular to colorectal cancer. Indeed, inflammatory bowel diseases like ulcerative colitis and colonic Crohn’s disease are strongly associated with colorectal cancer [6]. For example, inducing colitis to create chronic inflammation after introducing procarcinogen is an established and reliable two-step mouse model of colitis-associated cancer (CAC) [7,8,9].

Most common cancer treatments are designed to kill tumor cells. However, the way in which cells die is very important, because dying cells may release molecules that initiate an immune response. We shall refer to cells that go through the process of necrotic cell death as necrotic cells. Necrotic cells are known to release damage-associated molecular pattern (DAMP) molecules such as high mobility group box 1 (HMGB1), which triggers immune responses [10,11]. In particular, HMGB1 activates dendritic cells (DCs) [12]. There is evidence that the expressions of HMGB1 and RAGE, its receptor, are significantly higher in ulcerative colitis than in control cases [13]. HMGB1 has been observed in other cancers, as a result of treatments by radiotherapy and chemotherapy [12,14,15,16].

In colon cancer, activated CD8^+^ T cells enhance production of necrotic cells by expressing high levels of cytokines like IFN-γ and FasL [17]. Necrotic cells and macrophages release HMGB1 to activate dendritic cells [12], which leads to activation of T-cells [18]. In addition, intestinal epithelial cells, which are in close contact with DCs, activate dendritic cells by releasing molecules like thymic stromal lymphopoietin (TSLP) [19,20]. Once activated, dendritic cells release cytokines STAT4, STAT6, and IL-4, which induce differentiation of naive T-cells into effector T cells (Th1, Th17 and Th2) [21]. CD4${}^{+}$ T-cells can also become activated by TNF-α, which is released by $M_{1}$ macrophages [22]. Activated CD4${}^{+}$ T-cells release IL-2, 4, 5, 13 and 17 to activate killer cells like CD8${}^{+}$ T-cells [18,23,24]. CD4${}^{+}$ T-cells also release IFN-γ, which activates $M_{1}$ macrophages [25,26]. Activated macrophages and CD4${}^{+}$ effector T-cells release tumor-promoting cytokines interleukin 6 (IL-6) [27]. IL-6 promotes tumor growth by activating STAT3 in intestinal epithelial cells [28].

Knowledge of the cancer microenvironment is essential in predicting the progression of cancer. A strong correlation between in situ immune reactions in tumor regions and prognosis has been observed regardless of the local extent of the tumor and of invasion of regional lymph nodes [29]. A weak in situ immune reaction in tumor regions is associated with a poor prognosis even in patients with minimal tumor invasion (stage I). Moreover, high expression of the Th17 markers predicts a poor prognosis for patients with colorectal cancer, whereas patients with high expression of the Th1 markers have prolonged disease-free survival [30]. However, it has been observed that a high proportion of CD8${}^{+}$ T cells, effector memory T cells and CD4${}^{+}$ T cells is correlated with longer survival in colorectal cancer [31,32,33]. Moreover, in colon cancer, patients with a low level of macrophages have a deeper depth of invasion than patients with a high level of macrophages [33]. All these observations indicate the importance of the relative abundance of various immune cells, as well as their interaction networks, in the colonic tumors’ initiation and progression. Therefore, to accurately model the progress of cancer, we need to divide patients into similar cohorts based on their tumor-infiltrating immune cells and predict the progression for each group separately.

While there are many papers that use mathematical models for colon cancer progression [34,35,36,37,38,39,40,41,42,43], only a few have attempted to include immune interaction in their model. Models such as [40,41,42] define a system of ordinary differential equations (ODEs) that describe the interactions between cancerous cells and various sub-populations of immune cells (including NK cells, CD8+ T cells, lymphocytes, natural death cells and interleukins) and explore how these interactions can influence tumor growth over time. While time course data for the growth of untreated tumors are not currently easily available to verify models such as [40], other models such as [41] include simulations of treatment plans that can be compared on the population-level to results from previous clinical trials. To generate population-level simulation results while acknowledging the different responses to treatment that can arise from differing patient immune profiles, this study selects a range of parameter values to simulate 64 unique “virtual patients” for which to solve the system of ODEs describing potential treatments.

**Figure 1:**
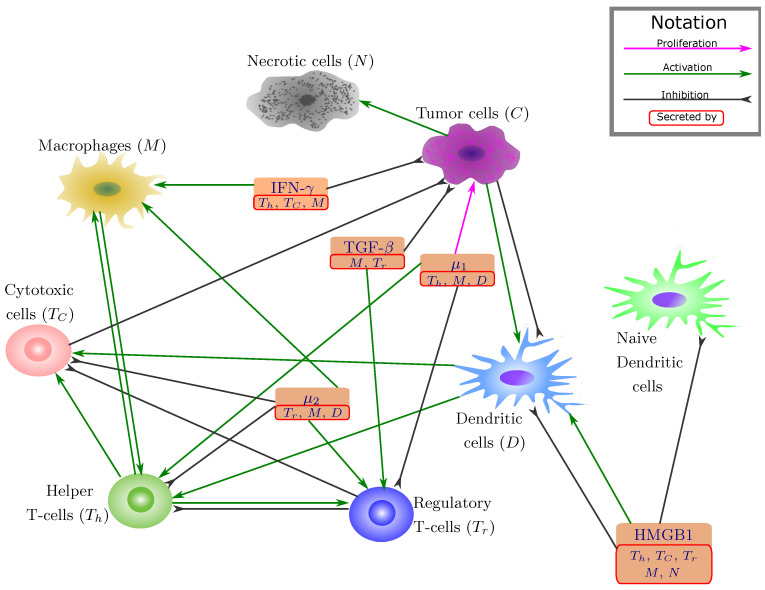
**Network of cells and cytokines**. Sharp arrows indicate activation or proliferation, and the blunt arrow indicates inhibitions.

In the present paper, we develop a data driven mathematical model of colon cancer with emphasis on the role of immune cells, including T-cells, dendritic cells and macrophages. Although there are many cell types and molecules involved in colon cancer, in order to avoid too much complexity, we only model some of the key players and interaction networks that we determined by reviewing articles on colon cancer progression. The resulting mathematical model is based on the network shown in Figure 1, and it is represented by a system of ODEs within the tumor. In order to explore differences in tumor growth among patients with different immune profiles, we use cancer patients’ data to estimate the percentage of each immune cell type in their primary tumors, and use clustering to group these patients into five distinct groups of immune patterns. We then use the data within each cluster to generate five “virtual patients”, for which we can calculate patient-specific parameters to use in the mathematical model. Lastly, we examine the differences in the resulting dynamics between the 5 clusters, and look for potential biomarkers that can link details of the tumor microenvironment to the dynamics of tumor growth.

## 2. Materials and Methods

### 2.1. Mathematical Model

We develop a mathematical model for colon cancer based on the interaction network among key players in colon cancer shown in Figure 1, and the list of variables is given in Table 1. The model is represented by a system of differential equations for concentrations and changing in time in unit of day. For clarity, we develop a simplified model in terms of ordinary differential equations. For biochemical processes A+B→C, we use the mass action law $\frac{dC}{dt}=\lambda AB$, where λ is production rate of *C* [44,45]. Throughout the paper, we use the symbol λ for production, activation or proliferation rates, and the symbol δ for decay, natural death or premature death (necrosis) rates.

#### 2.1.1. Cytokine Approximation

In order to reduce the complexity of the system, we treat some of cytokines as independent variables and approximate the value of other cytokines through already existing variables. Additionally, we combine the cytokines that have a similar function in the interaction network (Figure 1). Therefore, we combine IL-6, IL-17, IL-21 and IL-22 and denote their sum by the variable $\mu_{1}$. We also combine IL-10 and CCL20 and denote their sum by the variable $\mu_{2}$. The cytokines treated as model variables are HMGB1, IFN-γ, TGF-β, IL-6 and IL-10. We then model the dynamics of cytokines in the following way.

HMGB1 is passively released from necrotic cells [46], or actively secreted from activated T-cells and macrophages [47,48]. Thus, we can model the dynamics of HMGB1 by the equation:

(1) $$\frac{d\left[H\right]}{dt}=\lambda_{HN}\left[N\right]+\lambda_{HM}\left[M\right]+\lambda_{HT_{h}}\left[T_{h}\right]+\lambda_{HT_{C}}\left[T_{C}\right]+\lambda_{HT_{r}}\left[T_{r}\right]-\delta_{H}\left[H\right].$$

IL-6 is secreted by tumor associated macrophages (TAMs) [27,49,50,51], helper T-cells [27,51,52,53] and sub-population of dendritic cells [54,55]. IL-17, IL-21 and IL-22 are produced by helper T-cells [56]. Therefore, the resulting dynamics for $\left[\mu_{1}\right]$ can be written as

(2) $$\frac{d\left[\mu_{1}\right]}{dt}=\lambda_{\mu_{1}T_{h}}\left[T_{h}\right]+\lambda_{\mu_{1}M}\left[M\right]+\lambda_{\mu_{1}D}\left[D\right]-\delta_{\mu_{1}}\left[\mu_{1}\right].$$

IL-10 is produced by macrophages [57,58], dendritic cells [54,59] and T-reg cells [52,56,60,61]. CCL20 is produced by macrophages [62]. Thus, the equation for $\left[\mu_{2}\right]$ is

(3) $$\frac{d\left[\mu_{2}\right]}{dt}=\lambda_{\mu_{2}M}\left[M\right]+\lambda_{\mu_{2}D}\left[D\right]+\lambda_{\mu_{2}T_{r}}\left[T_{r}\right]-\delta_{\mu_{2}}\left[\mu_{2}\right].$$

IFN-γ is secreted by a sub-population of macrophages [57,63,64,65,66], helper T-cells [25,26] and cytotoxic cells [17], which results in the following equation:

(4) $$\frac{d\left[I_{\gamma }\right]}{dt}=\lambda_{I_{\gamma }T_{h}}\left[T_{h}\right]+\lambda_{I_{\gamma }T_{C}}\left[T_{C}\right]+\lambda_{I_{\gamma }M}\left[M\right]-\delta_{I_{\gamma }}\left[I_{\gamma }\right].$$

TGF-β is produced by macrophages [57,58] and T-reg cells [52,56,60,67] leading to the equation:

(5) $$\frac{d\left[G_{\beta }\right]}{dt}=\lambda_{G_{\beta }M}\left[M\right]+\lambda_{G_{\beta }T_{r}}\left[T_{r}\right]-\delta_{G_{\beta }}\left[G_{\beta }\right].$$

Other cytokines, like IL-2, IL-4, IL-5 and IL-13, we consider to be in a quasi-equilibrium state, i.e., proportional to the concentration of cells that secrete/produce them. In particular, IL-2, IL-5 and IL-13 are produced by CD4+ T-cells [18,24,68], so we consider

$$\begin{array}{c} \left[IL\text{-}2\right]\approx \mathrm{Const}\times \left[T_{h}\right],\\ \left[IL\text{-}5\right]\approx \mathrm{Const}\times \left[T_{h}\right],\\ \left[IL\text{-}13\right]\approx \mathrm{Const}\times \left[T_{h}\right]. \end{array}$$

IL-4 is also produced both by CD4+ T-cells [18,24,68] and dendritic cells [21], so we take

$$\left[IL\text{-}4\right]\approx \mathrm{Const}\times \left[T_{h}\right]+\mathrm{Const}\times \left[D\right].$$

IL-12 secreted by macrophages [57,58] and dendritic cells [49,50,54,56,69,70], thus can be approximated as

$$\left[IL\text{-}12\right]\approx \mathrm{Const}\left[M\right]+\mathrm{Const}\left[D\right],$$

while IL-23 and TNF-α are secreted solely by macrophages [49,50]; hence, their approximation is

$$\left[IL\text{-}23\right]\approx \mathrm{Const}\left[M\right],\;\left[TNF\text{-}\alpha \right]\approx \mathrm{Const}\left[M\right].$$

#### 2.1.2. T-Cells

In this model, we differentiate four subgroups of T-cells: naive, helper, cytotoxic and regulatory.

Naive T-cells, $T_{N}$, are not necessarily part of tumor microenvironment, as they usually are activated within lymph nodes. However, making activation rates for other types of T-cells proportional to the density of naive cells creates a better controlled system and avoids unlimited exponential growth. Thus, we summarize the equation for the dynamics of the naive T-cells after detailing the equations of other types of T-cells. The variables $T_{h}$, $T_{C}$ and $T_{r}$ correspond to the concentration of activated T-helper, cytotoxic and T-reg cells, respectively.

Helper T-cells can be activated with antigen presentation by dendritic cells [18]. CD4+ T-cells can be additionally activated by IL-12, while Th17 are activated by IL-6, TNF-α and IL-23 [56]. Regulatory T-cells inhibit protective immune response (helper and cytotoxic T-cells) in several ways including production of immunosuppresive cytokines such as IL-10 and CCL20 as well as through contact-dependent mechanisms [56]. Additionally, we introduce the natural death rate for helper cells $\delta_{T_{h}}$. The resulting equation is

(6) $$\frac{d\left[T_{h}\right]}{dt}=\left(\lambda_{T_{h}D}\left[D\right]+\lambda_{T_{h}M}\left[M\right]+\lambda_{T_{h}\mu_{1}}\left[\mu_{1}\right]\right)\left[T_{N}\right]-\left(\delta_{T_{h}\mu_{2}}\left[\mu_{2}\right]+\delta_{T_{h}T_{r}}\left[T_{r}\right]+\delta_{T_{h}}\right)\left[T_{h}\right].$$

The variable corresponding to cytotoxic cells accounts for the effects of cytotoxic T-lymphocytes (mainly CD8+ T-cells) and possibly natural killer cells. CD8+ T-cells are activated by IL-2, IL-4, IL-5 and IL-13 [18,24,68]. Cumulative effect of these cytokines can be written as

$$\left[IL\text{-}2,4,5,13\right]\approx \mathrm{Const}\times \left[T_{h}\right]+\mathrm{Const}\times \left[D\right].$$

Activation of natural killer cells requires IL-2 [71], which is already included. We also include inhibitory effects mediated by T-reg cells. The dynamics of $T_{C}$ cell group is modeled by the following equation:

(7) $$\frac{d\left[T_{C}\right]}{dt}=\left(\lambda_{T_{C}T_{h}}\left[T_{h}\right]+\lambda_{T_{C}D}\left[D\right]\right)\left[T_{N}\right]-\left(\delta_{T_{C}\mu_{2}}\left[\mu_{2}\right]+\delta_{T_{C}T_{r}}\left[T_{r}\right]+\delta_{T_{C}}\right)\left[T_{C}\right].$$

Regulatory T-cells can be activated by IL-2 [56,72,73], CCL20 [62] and TGF-β [56,67]. IL-6 suppresses T-reg differentiation and shifts it towards T-helper type [74]. The resulting dynamics can be described as follow:

(8) $$\frac{d\left[T_{r}\right]}{dt}=\left(\lambda_{T_{r}T_{h}}\left[T_{h}\right]+\lambda_{T_{r}\mu_{2}}\left[\mu_{2}\right]+\lambda_{T_{r}G_{\beta }}\left[G_{\beta }\right]\right)\left[T_{N}\right]-\left(\delta_{T_{r}\mu_{1}}\left[\mu_{1}\right]+\delta_{T_{r}}\right)\left[T_{r}\right].$$

Combining all activation and introducing independent naive T-cell production $A_{T_{N}}$, we get the following equation for naive T-cells:

(9) $$\begin{array}{cc} \frac{d\left[T_{N}\right]}{dt}= & A_{T_{N}}-\left(\lambda_{T_{h}D}\left[D\right]+\lambda_{T_{h}M}\left[M\right]+\lambda_{T_{h}\mu_{1}}\left[\mu_{1}\right]\right)\left[T_{N}\right]\\ \; & -\left(\lambda_{T_{C}T_{h}}\left[T_{h}\right]+\lambda_{T_{C}D}\left[D\right]\right)\left[T_{N}\right]\\ \; & -\left(\lambda_{T_{r}T_{h}}\left[T_{h}\right]+\lambda_{T_{r}\mu_{2}}\left[\mu_{2}\right]+\lambda_{T_{r}G_{\beta }}\left[G_{\beta }\right]\right)\left[T_{N}\right]\\ \; & -\delta_{T_{N}}\left[T_{N}\right]. \end{array}$$

#### 2.1.3. Dendritic Cells

Dendritic cells become activated by HMGB1 [12]. Moreover, TSLP, which is released by cancer cells [19,20], leads to the activation of dendritic cells (green arrow in Figure 1 from cancer cells to DCs). We take TSLP in quasi-equilibrium state as

$$\left[TSLP\right]\approx \mathrm{Const}\times \left[C\right].$$

On the other hand, multiple factors induced by cancer cells may promote natural death of dendritic cells [75,76,77,78,79] (black arrow in Figure 1 from cancer cells to DCs). Additionally, there’s evidence that HMGB1 can reduce the maturation rate of dendritic cells [61,79]. Introducing the independent production rate of naive dendritic cells $A_{D_{N}}$, we get the following system for dynamics of naive ($D_{N}$) and activated (*D*) dendritic cells:

(10) $$\begin{array}{cc} \frac{d\left[D_{N}\right]}{dt}= & A_{D_{N}}-\left(\lambda_{DH}\left[H\right]+\lambda_{DC}\left[C\right]\right)\left[D_{N}\right]-\left(\delta_{DH}\left[H\right]+\delta_{D}\right)\left[D_{N}\right], \end{array}$$

(11) $$\begin{array}{cc} \frac{d\left[D\right]}{dt}= & \left(\lambda_{DH}\left[H\right]+\lambda_{DC}\left[C\right]\right)\left[D_{N}\right]-\left(\delta_{DH}\left[H\right]+\delta_{DC}\left[C\right]+\delta_{D}\right)\left[D\right]. \end{array}$$

#### 2.1.4. Macrophages

There are two main sub-types of macrophages: M1 and M2. M1 phenotype can be activated by IFN-γ, while M2 can be activated IL-4 and IL-13, which are secreted by helper T-cells [57,58]. Additionally there’s a possibility of TAM activation by IL-10 [57,80,81]. Introducing naive ($M_{N}$) and activated (*M*) TAMs, as well as production rate for naive macrophages $A_{M}$, we can write the following system:

$$\begin{array}{cc} \frac{d\left[M_{N}\right]}{dt}= & A_{M}-\left(\lambda_{M\mu_{2}}\left[\mu_{2}\right]+\lambda_{MI_{\gamma }}\left[I_{\gamma }\right]+\lambda_{MT_{h}}\left[T_{h}\right]\right)\left[M_{N}\right]-\delta_{M}\left[M_{N}\right],\\ \frac{d\left[M\right]}{dt}= & \left(\lambda_{M\mu_{2}}\left[\mu_{2}\right]+\lambda_{MI_{\gamma }}\left[I_{\gamma }\right]+\lambda_{MT_{h}}\left[T_{h}\right]\right)\left[M_{N}\right]-\delta_{M}\left[M\right]. \end{array}$$

It has been shown that there is a diverse spectrum of TAM sub-types [82]. We therefore decided to combine all activated macrophages, including both M1 and M2 macrophages into one variable *M* and let the system parameters carry the differences between sub-populations and determine which effects prevail.

Next, to simplify the system, we introduce the total amount of macrophages $M_{0}=\left[M_{N}\right]+\left[M\right]$. Adding the above equations, we get $\frac{dM_{0}}{dt}=A_{M}-\delta_{M}M_{0}.$ If we assume initial conditions for $M_{0}$ to be at the equilibrium $M_{0}=A_{M}/\delta_{M}$, then $M_{0}$ will remain constant at all times. Then we can express naive macrophages as $\left[M_{N}\right]=M_{0}-\left[M\right]$ and write the resulting equation for macrophages as follows:

(12) $$\frac{d\left[M\right]}{dt}=\left(\lambda_{M\mu_{2}}\right.\left[\mu_{2}\right]+\lambda_{MI_{\gamma }}\left[I_{\gamma }\right]\left.+\lambda_{MT_{h}}\left[T_{h}\right]\right)\left(M_{0}-\left[M\right]\right)-\delta_{M}\left[M\right].$$

#### 2.1.5. Cancer Cells

Cancer cells are epithelial cells with abnormally high growth and abnormally small death rate. Additional loss of apoptosis (cell death) in cancer cells is induced by IL-6 [75,77,83,84]. In addition to innate abnormally high proliferation rate $\lambda_{C}$, proliferation in cancer can be stimulated by expression of STAT3 in cancer cells, where STAT3 is activated by cytokines such as IL-6, IL-17, IL-21 and IL-22 [56,85]. On the other hand, cancer development is suppressed by TGF-β [56,86,87,88], IL-12 and IFN-γ [56]; the suppressive properties of IL-12 are mediated by IFN-γ [89] (so it is not directly included in the equation). Cytotoxic T-cells also directly target cancer cells for destruction [56]. In cancer modeling, proliferation is traditionally taken to be proportional to $\left[C\right]\left(1-\left[C\right]/C_{0}\right)$, where $C_{0}$ is the total capacity [90,91]. Effect of dendritic cells on cancer cells is only modeled through intermediary agents, such as T-cells, IL-6 and IL-10. Thus, the resulting equation is

(13) $$\frac{d\left[C\right]}{dt}=\left(\lambda_{C}+\lambda_{C\mu_{1}}\left[\mu_{1}\right]\right)\left[C\right]\left(1-\frac{\left[C\right]}{C_{0}}\right)-\left(\delta_{CG_{\beta }}\left[G_{\beta }\right]+\delta_{CI_{\gamma }}\left[I_{\gamma }\right]+\delta_{CT_{C}}\left[T_{C}\right]+\delta_{C}\right)\left[C\right].$$

#### 2.1.6. Necrotic Cells

We designate cells which go through the process of necrotic cell death as necrotic cells. Since there is a limited amount of resources in the tumor microenvironment, and cells are under pressure, there are always some necrotic cells produced by the tumor. In addition, when activated cytotoxic T-cells kill colorectal cancer cells by expressing high levels of cytokines like IFN-γ and FasL [17], a fraction of the cancer cells may go through the stage of first becoming necrotic cells. Therefore, the rate of “production” of the necrotic cells is given by the fraction of dying cancer cells ($\alpha_{NC}$) and the resulting dynamics can be written as follows:

(14) $$\frac{d\left[N\right]}{dt}=\alpha_{NC}\left(\delta_{CG_{\beta }}\left[G_{\beta }\right]+\delta_{CI_{\gamma }}\left[I_{\gamma }\right]+\delta_{CT_{C}}\left[T_{C}\right]+\delta_{C}\right)\left[C\right]-\delta_{N}\left[N\right].$$

### 2.2. Non-Dimensionalization and Sensitivity Analysis

For additional numerical stability and to eliminate scale dependence, we perform non-dimensionalization of the system. For variable *X* converging to a steady-state $X_{\infty }$, we consider non-dimensional variable $\bar{X}=X/X_{\infty }$. Then, $\bar{X}$ satisfies the equation

$$\frac{d\bar{X}}{dt}=F\left(\bar{X},\;\theta ,\;t\right).$$

The (first order) solution sensitivity *S* with respect to the model parameter $\theta ={\left\{\theta_{i}\right\}}_{i=\bar{1,\;N}}$ is defined as a vector

$$S_{i}=\frac{d\bar{X}}{d\theta_{i}},\;i=\bar{1,\;N}.$$

In general, the sensitivity vector is time dependent and varies for different solutions and parameter sets [92,93,94]. However, here we consider sensitivity at the steady-state of the equation. The sensitivity of each parameter in the neighborhood of a chosen parameter set Ω(θ) is defined as

$$S_{i}=\int_{\Omega }S_{i}\left(\theta \right)\;d\theta ,$$

where the integration is evaluated numerically with sparse grid points [95,96].

We choose three quantities of interest for the sensitivity analysis: amount of cancer cells *C*, total amount of cells, and a measure of how fast the system is converging to the steady-state. Consider general steady-state system as follows

$$F(X^{★},\;\theta )=0,$$

where $X^{★}$ is the equilibrium. We then consider a small perturbation to $X^{★}$ as $\bar{X}\left(t\right)=X^{★}+\varepsilon X_{1}\left(t\right)$. The linearized system becomes

$$\frac{dX_{1}\left(t\right)}{dt}=\nabla F(X^{★},\;\theta )X_{1}\left(t\right)+O\left(\varepsilon \right),$$

where $\nabla F\left(X,\;\theta \right)$ is the Jacobian matrix of $F\left(X,\;\theta \right)$ with respect to *X*. Thus, we have $X_{1}\left(t\right)\approx e^{\nabla F(X^{★},\;\theta )t}$ and the minimal eigenvalue $\min\lambda (\nabla F(X^{★},\;\theta ))$ determines how fast it reaches the steady-state.

### 2.3. Cancer Patients’ Data

In recent years, several tumor deconvolution methods have been developed to estimate the relative abundance of various cell types in a tumor from its gene expression profile. A review of these methods [97] and an application of CIBERSORTx on renal cancer [98] show a great performance of CIBERSORTx model. To identify the immune profiles of colonic tumors, we applied CIBERSORTx [99] on RNA-seq gene expression profiles of primary tumors of patients with colon cancer from the Cancer Genome Atlas (TCGA) project of Colon Adenocarcinoma (COAD) downloaded from University of California Santa Cruz (UCSC) Xena web portal. There are a total of 329 patients with RSEM normalized RNA-seq data in $\log_{2}$ scale. Before applying CIBERSORTx on this data set, we transformed the gene expression values to the linear space.

### 2.4. Numerical Methods

In order to solve the time dependent system, we employ the SciPy odeint function [100] using initial conditions based on patients with the smallest tumor area within each cluster. The sensitivity analysis of the system based on the cancer and total cell density at steady-state is obtained analytically by differentiating the steady-state equation with respect to the parameters, namely,

$$\nabla F(X^{★},\;\theta )\frac{dX^{*}}{d\theta }+\frac{\partial F(X^{★},\;\theta )}{\partial \theta }=0.$$

Then to obtain the sensitivity, $\frac{dX^{*}}{d\theta }$, one just needs to numerically invert the matrix ∇F. On the other hand, it is hard to analytically obtain the sensitivity of the eigenvalue, so instead a finite-difference approach is used as follows:

$$\frac{d\min\lambda (\nabla F(X^{★},\;\theta ))}{d\theta }\approx \frac{\min\lambda \left(\nabla F\left(X^{★},\;\theta +\frac{1}{2}\Delta \theta \right)\right)-\min\lambda \left(\nabla F\left(X^{★},\;\theta -\frac{1}{2}\Delta \theta \right)\right)}{\Delta \theta },$$

where Δθ is a small discretization parameter.

## 3. Results

We derived an ODE system describing complex dynamics in the colon cancer microenvironment. Assuming non-negative values of all parameters and non-negative initial conditions, the solution of the system remains non-negative and globally bounded (see Appendix A).

### 3.1. Patient Data Analysis

**Figure 2:**
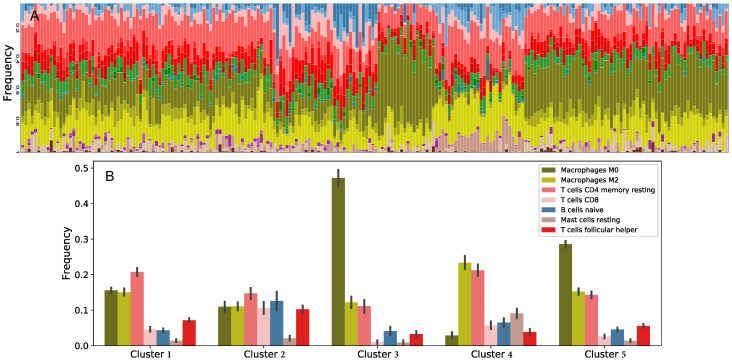
**Immune cell fractions.** (**A**) The fraction of immune cells in each colonic tumor. (**B**) The frequencies of immune cell types in each cluster of patients. Clusters were formed based on variations in 22 immune cell types, some of which were later combined and others that were not included in the model. Cell frequencies in this figure are averaged within the cluster. The vertical bars show the standard deviations.

We downloaded TCGA clinical data, which includes tumor dimension, stage, gender, vital and tumor status at last follow up, as well as gene expression profiles of primary tumors for patients with colon cancer from the Genomic Data Commons (GDC) portal. We applied CIBERSORTx B-mode on gene expression profiles to estimate the fraction of each immune cell type in each tumor. Elbow method applied on estimated cell fractions (Figure 2A) showed the existence of five distinct immune patterns. We hence performed K-means clustering with K=5, in order to group patients based on the immune pattern of their primary tumors. Figure 2B shows average cell fractions for the patients in each of the five clusters. In order to demonstrate the immune variation between these clusters, the average frequencies shown in this plot are of immune cells that have high discrepancy in abundance between clusters. To investigate the effect of these immune patterns on the dynamics of tumors, we model each cluster separately, and based on the steady-state assumptions (see Appendix B). We assume that tumors in each cluster might behave differently not only because of immune cell variations, but also because of variations in their parameter values. For this reason, we estimate parameter values for each cluster separately based on steady-state values derived from patient data, as described further below. The effects of variations in parameter values are investigated through sensitivity and dynamic analyses.

The deconvolution data, described in Section 2.3, only provide the ratios of immune cells in the tumor microenvironment. These data are utilized to obtain the values of variables as detailed in Appendix C. For each patient *P*, we define their size of tumor, size(P), to be the product of the longest and the shortest dimensions of the tumor, and we assume total cell density is proportional to the size of the tumor:

$$\mathrm{Total}\_\mathrm{Cell}\_{\mathrm{Density}}_{P}=\alpha_{dim}\frac{\mathrm{size}(P)}{\frac{1}{K}\sum_{\mathrm{all}\;P}\mathrm{size}\left(P\right)}.$$

Then, we take each immune cell value from deconvolution multiplied by $0.4\alpha_{dim}\sum \left(\mathrm{Immune}\;\mathrm{cell}\;\mathrm{ratios}\right)$ and

$$C=\frac{2}{3}\left(\mathrm{Total}\_\mathrm{Cell}\_\mathrm{Density}-\mathrm{Total}\_\mathrm{Immune}\_\mathrm{Density}\right),\;N=0.5C.$$

while macrophage capacity $M_{0}$ is derived from the data, we assume cancer capacity to be $C_{0}=2*C$ for both mean-based and extreme-based data. We choose the scaling factor $\alpha_{dim}=1.125\times 10^{5}$ to approximately match the average density of cancer cells across all patients to $4.5\times 10^{4}$ cells/cm^3^ reported in [101]. However, it is important to note that this is no more than scaling and has no effect on the dynamics of the dimensionless system.

**Figure 3:**
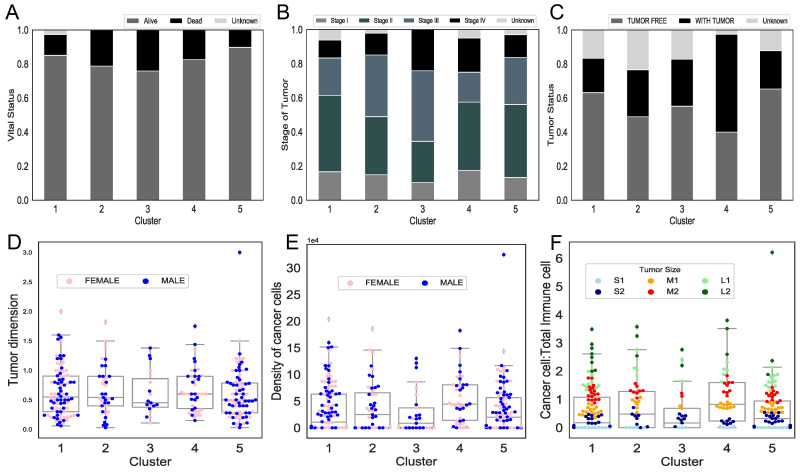
**Clinical features of the clusters.** (**A**–**C**) The percentage of patients alive or dead at the last time of follow up (**A**), the stage of tumors I-IV at time of initial diagnosis (**B**) and tumor status (with tumors or without tumors) at the last time of follow up (**C**). (**D**–**F**) Tumor dimension (**D**), density of cancer cells (**E**) and ratio of cancer to total immune cells (**F**) in each cluster, respectively. Colors in (**F**) show the different tumor dimensions, grouped into six categories (cm^2^): S1: 0–0.25, S2: 0.25–0.5, M1: 0.5–0.75, M2: 0.75–1, L1: 1–1.25, L2: >1.25.

We further investigate the clinical features of the clusters to see if there are other differences between clusters rather than ratio of immune cells. Although distributions of gender (Figure 3E,F), tumor dimension (multiplication of the longest and the shortest dimension) (Figure 3D), density of cancer cells, and ratio of cancer to immune cells (Figure 3F) show similar trends in each cluster, we observed some differences in clinical outcomes between the clusters. For example, cluster 5 has the highest percentage of alive patients and tumor-free patients, while cluster 4, which has the lowest M0 macrophages and the highest M2 macrophages, has the highest percentage of patients with tumor at the last time of follow up. In addition, a Chi-squared test shows that cluster 4 has significantly different percentage of tumor status compared to clusters 1 and 5 with *p*-values 0.0002 and 0.0006, respectively. In addition to these, cluster 3, which has the highest frequency of M0 macrophages, has the highest proportion of deceased patients and stages III and IV tumors. These observations suggest that these clusters represent different immune patterns that might lead to different outcomes. Importantly, no immune cell has a correlation higher than 0.6 with any other immune cells or cancer cells in each cluster. Moreover, Figure 3F demonstrates that as the size of a tumor increases, the ratio of cancer cells over the total number of cells increases.

For each cluster, we consider the mean of variables of patients with tumor size above the average of their cluster as the steady-state values of the variables for the corresponding cluster. The resulting data are given in Table 2. These data are only used as in Appendix B to generate the parameter sets given in Table A2 and Table A3. In other words, we assume the largest tumors in each cluster are near their steady-state. Since we have estimated the values of all variables for all tumors from their gene expression profiles, we have the values of all variables at the steady-state. To estimate the parameters, we first set the left side of the ODEs to zero, in order to create a system of equations that does not depend on time. In order to obtain unique solutions, we need to have the same number of equations as the number of parameters. Therefore, we estimate a small number of parameters using biological studies. We also assume some relations among parameters as described in Appendix B. Hence, by these assumptions, we obtain a unique set of parameter values for each cluster, and therefore all parameters are identifiable.

In order to investigate the dynamics of tumors and solve the system of ODEs, we need the initial values of variables. We assume the smallest tumors in each cluster represent the initial conditions. Therefore, we use the values of the variables of the smallest tumors (estimated using their gene expression profiles) as the initial conditions. The relative values are given in Table 3. The dynamics with initial conditions based on other patients are presented in Appendix C.

### 3.2. Sensitivity Analysis

**Figure 4:**
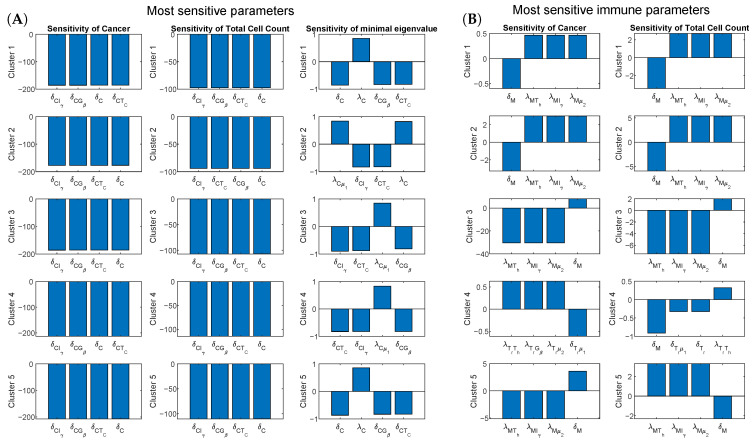
**Sensitivity analysis**. The first, second and third columns of (**A**) respectively present the results of non-dimensional sensitivity of cancer cell density, total cell density and minimal eigenvalue of the Jacobian of the system at the steady-state. Minimal eigenvalue is used as a measure of how fast the system converges to the steady-state. (**B**) The sensitive parameters related to immune cells. Each row of plots shows the most sensitive parameters for each cluster of patients.

We perform sensitivity analysis of the non-dimensionalized system with parameters derived from patient data through steady-state assumptions. Table 2 contains the steady-state values used for each cluster, and Appendix B shows the parameter derivation and non-dimensionalization in detail. It is worth pointing out that there might be a variation in the calculated parameters due to differences between patients and possible alterations in assumptions of Appendix B. In order to account for those possible variations, sensitivity analysis is performed on dimension-less system of equations (see Section 2.2). We use cancer cells, total cell density and minimal eigenvalue of the Jacobian of the ODE system as the variables of interest in the sensitivity analysis. Minimal eigenvalue of the Jacobian serves as a measure of how fast the system converges to the steady-state. Figure 4A shows the four most sensitive parameters for each cluster. Additionally, to evaluate the effect of immune microenvironment on cancer, we look at the sensitivity of cancer cells and total cell density excluding the parameters appearing in the equations for cancer and necrotic cells. The resulting data denoted as “Immune sensitivity” are given in Figure 4B.

Across all clusters, the most sensitive parameters are cancer proliferation and death rates directly present in the cancer Equation (13). From the third column in Figure 4A, we conclude that for all clusters, increased cancer proliferation coefficients correspond to faster convergence to the steady-state, while increased cancer death rates lead to a slower convergence. When considering immune sensitivity presented on Figure 4B, in clusters 1, 2, 3 and 5, the most sensitive immune parameters are those corresponding to the activation and decay rates of macrophages, with only sensitivity levels being different between clusters and variables. In clusters 1 and 2, which include tumors with a smaller density of naive macrophages than activated macrophages, an increase in decay rate of macrophages causes a decrease in the density of cancer cells and total cell density. On the other hand, an increase in any of the activation rates for macrophages causes an increase in both quantities of interest. However, for clusters 3 and 5, which include tumors with a higher density of naive macrophages than activated macrophages, the effects are reversed. Interestingly, for cluster 3, the increase in macrophage activation rate results in both lower cancer cell density and total cell density, with latter sensitivity being noticeably smaller by absolute value. On the other hand, for cluster 5 the increase in macrophage activation rate results in lower cancer cell density, but higher total cell density. This can be explained by a significant increase in immune cell density, which for cluster 5 is even higher than the corresponding decrease in cancer cell density. All these results demonstrate that at the steady-state, tumor-associated macrophages could have different effects on different clusters of patients depending on their immune profile.

The outlying cluster 4, which consists of tumors with a significantly small density of naive macrophages compared to the other clusters, is less sensitive to the activation rates of macrophages. The most sensitive immune parameters for cancer cell density are those related to the activation and degradation of regulatory T-cells. The results indicate that increased regulatory response activation rate corresponds to an increase in the cancer cell density, while an increase in T-reg cell degradation rate results in a decrease in cancer cell density, demonstrating that for this cluster of patients regulatory T-cells have mostly negative effects. Importantly, the most sensitive parameter for the total cell density is still the decay rate of macrophages, and macrophages still have a negative effect, i.e., the faster decay of macrophages leads to the smaller tumors in the steady-state.

### 3.3. Dynamic of Tumor Microenvironment

**Figure 5:**
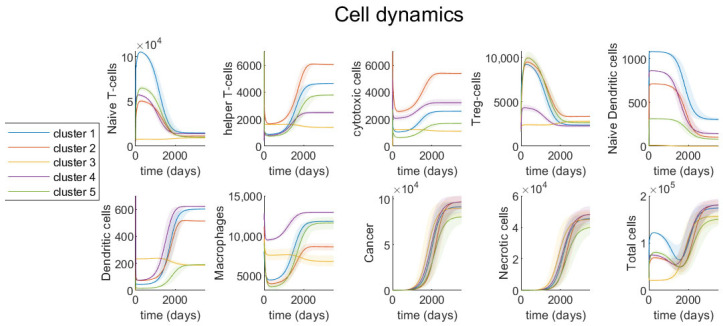
**Cells’ dynamics in colonic tumors**. Time evolution of cells’ density (cell/cm^3^) for each cell type in the model and total cell density. Different colors represent the models derived for different clusters of patients and shaded regions represent the 10% variation in the most sensitive parameters.

**Figure 6:**
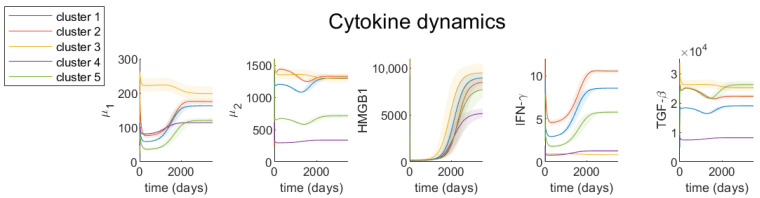
**Cytokines’ dynamics in colonic tumors**. Time evolution of RNA-seq expression rate of cytokines. Different colors represent the models derived from different clusters of patients and shaded regions represent the 10% variation in the most sensitive parameters.

We investigate the dynamics of each variable, with parameters derived for each cluster based on steady-state assumptions (see Table 2 for steady-state values and Table A1, Table A2, Table A3 for parameter values) and initial conditions of patients with the smallest tumor (see Table 3). Figure 5 and Figure 6 show the dynamics of cell densities and cytokines expressions respectively. We investigate the effect of variations in parameters’ values on the dynamics of the tumor by varying the most sensitive parameters by 10% in both the positive and negative directions. These variations are shown as shaded regions on each of the graphs.

For most clusters, cancer cells grow as helper T-cells, cytotoxic cells (cytotoxic T-cells and NK cells), dendritic cells and macrophages increase in density over time, while naive T-cells, regulatory T-cells and naive dendritic cells decrease in density. The increase in cytotoxic cells along with tumor progression is somewhat contradicting to the finding in [102,103] that colon primary tumor growth is associated with decreased cytotoxic T-cells density. However, there is no correlation between tumor size and cytotoxic cells in the TCGA data of colonic primary tumors. Moreover, it is important to note that in our model cancer cells’ growth is multiple times faster than the rate of change of any immune cells (Figure 5). Thus, even though cytotoxic cells density grows over time, the tumor is growing at a much faster rate. Since tumor cells activate dendritic cells which then activate cytotoxic cells, it is reasonable to see some growth of cytotoxic cells when tumor cells density increases rapidly.

Clusters 2 and 4 have the highest cancer cell density at steady-state and also the highest growth rate of cancer cells. Cluster 2’s cancer cells start out with the lowest growth rate, but at around 1800 days grow significantly faster and end up growing the fastest among all clusters. Cluster 2 has the highest density of helper T-cells and cytotoxic cells, both in the early stages of cancer development and at steady-state, as well as the highest growth rate of these cells. However, cluster 2 has rather low density and low growth rate of macrophages.

Cluster 4, having the largest density of activated macrophages and a significantly small density of naive macrophages (Figure 2B), first demonstrates average cancer growth rate, but then increases and has one of the two highest cancer cell densities at the steady-state (Figure 5). Similar to cluster 2, cluster 4 has high density of cytotoxic cells (CD8 T-cells and NK cells) initially and at steady-state. Both clusters 2 and 4 have a low growth rate of macrophages and a high density of dendritic cells, compared to other clusters. Immune cell dynamics of clusters 2 and 4 demonstrate that a high density of cytotoxic cells and dendritic cells, along with a low growth rate of macrophages, correlates with a high growth rate of cancer cells.

However, unlike cluster 2, cluster 4 has a low growth rate of cytotoxic cells and helper T-cells, and a low density of helper T-cells overall. Though both clusters 2 and 4 have a low growth rate of macrophages, cluster 4 has the highest density of macrophages among all clusters, while cluster 2 has the second lowest macrophages density. Regulatory T-cells also behave very differently between cluster 2 and cluster 4. Cluster 2 has high density and high decline rate of regulatory T-cells over time, but cluster 4 has both low density and low decline rate of this cell. These observations suggest that cell densities alone cannot predict cancer progression and there are no specific biomarkers that are sufficient to model tumor growth. Instead, a time series immune interaction network with tumor cells can be useful in modeling cancer development.

Cluster 5, with the density of activated macrophages being slightly less than naive macrophages (Figure 2B), has the lowest cancer cell density at steady-state and the lowest cancer cell growth of all clusters (Figure 5). This cluster has the lowest growth rate and density at initial condition and steady-state of naive dendritic cells, activated dendritic cells and cytotoxic cells, except for cytotoxic cells density at steady-state (second lowest). It also has the highest growth rate of macrophages among the five clusters. This observation might imply that slow tumor growth is associated with low density and growth rate of naive and activated dendritic cells, cytotoxic cells and high growth rate of macrophages.

Cluster 1, which is characterized by the second largest population of macrophages and helper T-cells, demonstrates that dendritic cells alone cannot be chosen as a marker of cancer progression, as it has the second highest dendritic cell population, but only the third highest cancer cell population at the steady-state, being surpassed by cluster 2.

Cluster 3, being a clear outlier in the immune dynamics, has near zero density of naive dendritic cells. This alone prevents it from creating significant variations in the immune response during the cancer progression. It is interesting to note, that while almost unchecked by immune responses, this cluster initially demonstrates noticeably highest cancer growth rate, but results in the second lowest cancer density at the steady-state.

Tumor cytokines’ dynamics (Figure 6) indicate that as the tumor grows, HMGB1, IFN-γ and $\mu_{1}$ (IL-6, IL-17, IL-21, IL-22) increase in density, but TGF-β and $\mu_{2}$ (IL-10, CCL20) stay relatively constant. Clusters 2 and 4, which have the highest cancer cell growth rate among all clusters, show different cytokines’ behaviors throughout time. At steady-state, cluster 4 has significantly lower densities of all cytokines in our model than cluster 2, despite the fact that they have the same cancer cell density then. Cluster 4 also has a much lower growth rate of $\mu_{1}$, HMGB1 and IFN-γ compared to cluster 2. Clusters 1 and 5 have more similar growth rates of these cytokines as cluster 2, even though they have rather different tumor growth rates from cluster 2. Thus, the density or growth of any specific cytokine is not an adequate predictor of tumor progression, and we need the full interaction network to effectively model the cancer cell growth.

Additionally within each cluster, we look at the dynamics of cancer and total cell density with different initial conditions, each derived from a different patient in that cluster. See Appendix C, and specifically Figure A2, Figure A3, Figure A4, Figure A5, Figure A6, for more details on different initial conditions and resulting dynamics. This result indicates that even within the same cluster, different initial immune profiles may cause dramatic differences in the cancer progression rate. Additionally, while the dynamics of cancer cell density remains monotone across all patients, we observe oscillatory behavior in the total cell density. This can be explained by a temporary surge of immune cell density at the early stages of cancer, which also appears to correlate with slower cancer progression rate. The only cluster which does not exhibit this oscillatory behavior is cluster 3. As mentioned before, due to lack of naive dendritic cells, cluster 3 does not show significant immune cell density variations, which are the source of oscillatory behavior for other clusters.

## 4. Discussion

Although there are many mathematical models for cancer [34,35,36,37,38,39,40,41,42,43,104,105,106,107,108,109,110,111,112,113,114,115,116,117,118,119,120,121,122,123,124], one of the outstanding challenges in mathematical modeling of cancers is the existence of many unknown parameters and the limited number of data sets. For this reason, the approach of many of these mathematical models is to assume some values for parameters, use estimated parameters from other diseases, or vary the parameters and initial conditions within biologically-feasible values in order to investigate their effects on the results. Here, we choose some of the parameter values based on biological studies and the rest by utilizing patients’ gene expression data. New advances in tumor deconvolution techniques help us to utilize cancer patients’ data in order to develop a data driven mathematical model of tumor growth. Using tumor deconvolution methods, we estimate the relative abundance of various cell types from gene expression profiles of tumors. The machine learning algorithm of K-means clustering indicates the existence of five distinct groups of colon cancers based on their immune patterns. The comparison of tumor behaviors in these groups suggests that the dynamics of tumors strongly depends on their immune structure.

**Figure A1:**
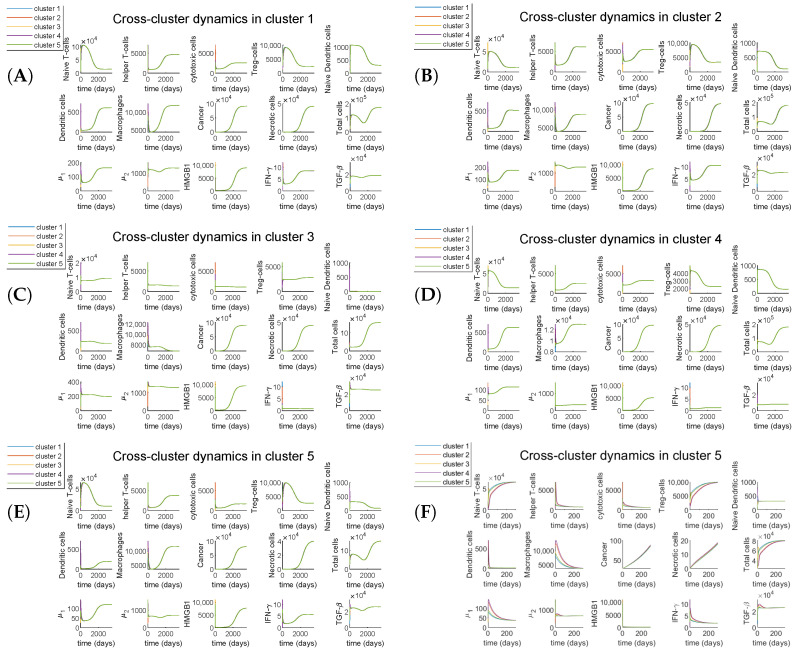
**Cross-cluster dynamics**. (**A**) Dynamics with parameters from cluster 1 and initial conditions from all clusters. (**B**) Dynamics with parameters from cluster 2 and initial conditions from all clusters. (**C**) Dynamics with parameters from cluster 3 and initial conditions from all clusters. (**D**) Dynamics with parameters from cluster 4 and initial conditions from all clusters. (**E**) Dynamics with parameters from cluster 5 and initial conditions from all clusters. (**F**) Initial dynamics with parameters from cluster 5 and initial conditions from all clusters on a time interval [0, 300].

While it would be ideal to use time course gene expression data of colon cancer patients in our framework, the availability of these time series data sets is limited. In order to combat this limitation, clustering was used to group patients with similar immune patterns and treat each group as time course data based on the size of tumor, which means the data points with small tumor density are considered data from early stages (initial conditions) and the data points with large tumor density are considered data from late stages (steady-state values). We assume a large tumor in a cluster that is in the steady-state is an evolution of a small tumor in the same cluster, because when a small tumor in a cluster (e.g., 1) evolves to a large tumor in another cluster (e.g., 3), its dynamics will quickly converge to the dynamics of a small tumor in the cluster of the steady-state tumor (e.g., 3) (Figure A1). We then follow a common approach of mathematical biology models that use assumptions on the steady-state values of the system to estimate parameters of the model [125,126]. Note that we use patient data to estimate the steady-state values of each cluster, and we then estimate parameters based on the values at the steady-state. Due to the non-dimensionalization process, the relative dynamics of the system are independent of the data scaling and only depend on relative values for patients.

**Figure A2:**
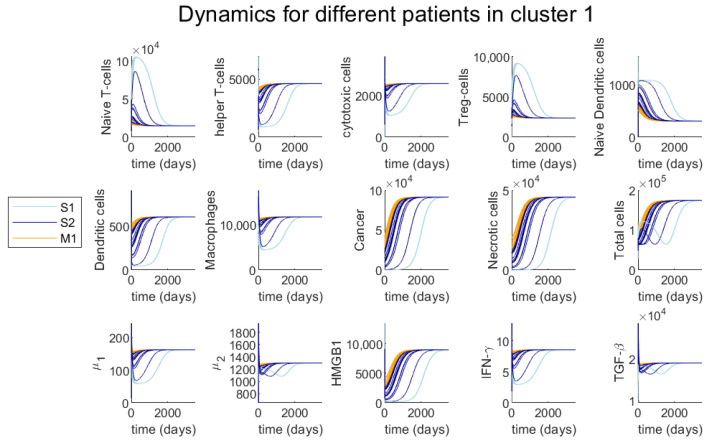
**Different initial conditions for cluster 1**. Based on patients in the “below average” category. Colors correspond to tumor size categories introduced in Figure 3F.

**Figure A3:**
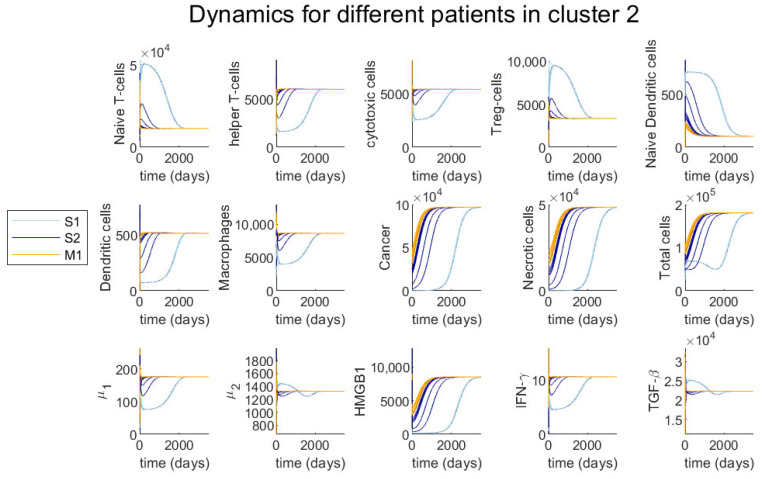
**Different initial conditions for cluster 2**. Based on patients in the “below average” category. Colors correspond to tumor size categories introduced in Figure 3F.

**Figure A4:**
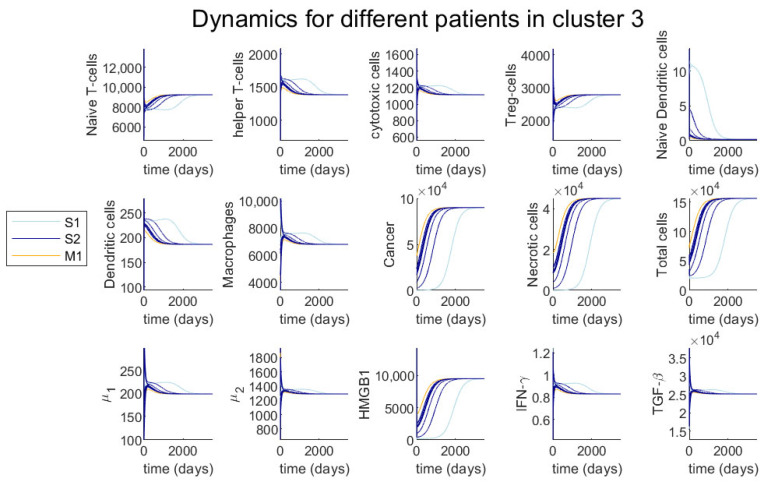
**Different initial conditions for cluster 3**. Based on patients in the “below average” category. Colors correspond to tumor size categories introduced in Figure 3F.

**Figure A5:**
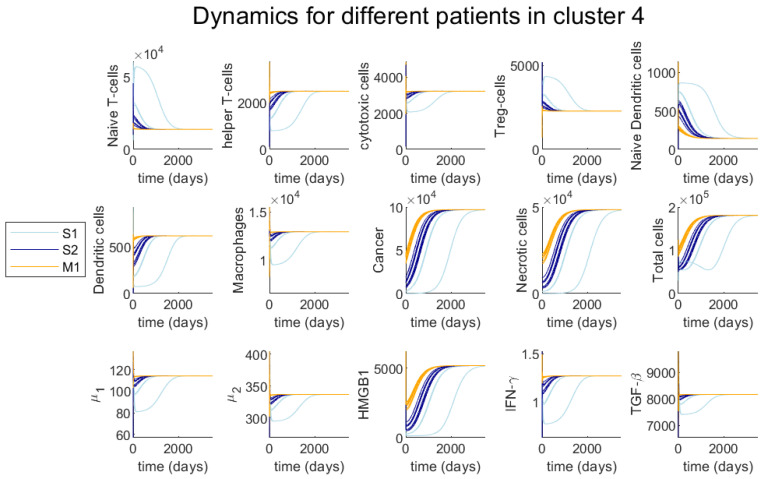
**Different initial conditions for cluster 4**. Based on patients in the “below average” category. Colors correspond to tumor size categories introduced in Figure 3F.

**Figure A6:**
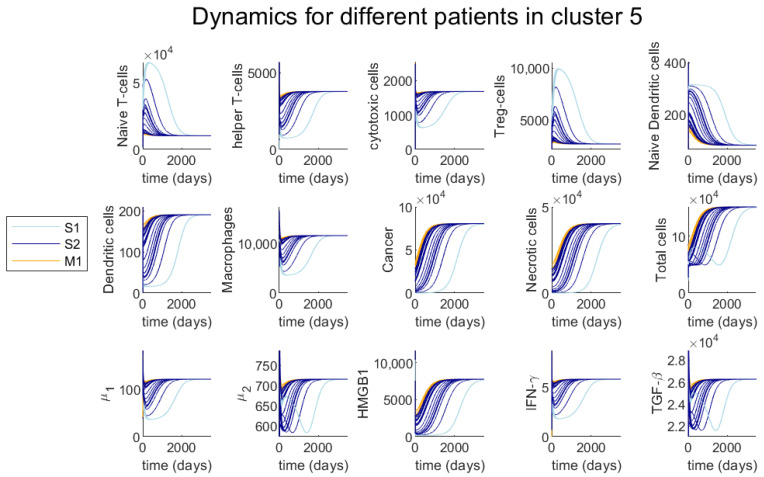
**Different initial conditions for cluster 5**. Based on patients in the “below average” category. Colors correspond to tumor size categories introduced in Figure 3F.

The parameter values have been estimated based on the assumption that the largest tumors are near the steady-state, because the large tumors do not have much space to grow. Figure 3F shows that the ratio of cancer cells over total immune cells increases when the size of tumors increases. This may suggest that small tumors have a broader potential for decision making and more options to evolve than large tumors. We also investigate the dynamics of all tumors with a size “below average” as initial condition. The resulting dynamics (Figure A2, Figure A3, Figure A4, Figure A5, Figure A6) show that when the size of initial tumors increases, the time to reach the steady-state decreases. This observation is also in support of the steady-state assumption.

To evaluate the effect of each parameter value on the results, we perform a comprehensive sensitivity analysis, which covers a range of parameter values. For all parameters that have not been determined using biological literature, we estimate 5 different values for each parameter in each cluster. We find the most sensitive parameters by applying a gradient based sensitivity analysis method on the dimension-less system of equations. Note, although we used completely different values for some of the parameters for each cluster, our sensitivity analysis shows that a similar set of parameters are the most sensitive parameters for all clusters. This demonstrates that while many parameters are unknown, the evolution of tumors is not sensitive to many of these parameters (Figure 4). For those sensitive parameters, we show how their variations would affect the results of the model (Figure 5 and Figure 6).

The mathematical model shows a unique pattern of tumor growth in each cluster based on their immune infiltration. For example, the model indicates that a high density of cytotoxic T-cells and dendritic cells and a low growth rate of macrophages are associated with a high growth rate of cancer cells (observed in clusters 2 and 4, Figure 4 and Figure 5), while a low density and growth rate of naive and active dendritic cells and cytotoxic T-cells and a high growth rate of macrophages correlate with slow tumor growth (observed in cluster 5, Figure 4 and Figure 5). Clinical information provided for patients in the clusters also supports the results of the dynamical model. Cluster 4, which has the highest percentage of patients with tumors at the time of last follow up (Figure 3C) has poor outcome compared to other clusters, while cluster 5, which has the highest percentages of tumor-free and alive patients (Figure 3A,C) has better outcome than the other clusters.

Importantly, our results imply that macrophages’ activation rates have different effects in different clusters. A high activation rate of macrophages leads to a high density of cancer cells in clusters 1 and 2 (Figure 4), in which there are more activated than naive macrophages. This result is in agreement with an observation of [127], which indicates a high CD206/CD68 ratio is significantly associated with poor outcome. Note, CD206 is the marker for M2 macrophages and CD68 is expressed at high levels on M0 cells [128]. However, the activation rates of macrophages are negatively correlated with tumor growth in clusters 3 and 5 (Figure 4), in which they are more naive than activated macrophages. This is consistent with the observation that a high level of macrophages is associated with a favorable outcome of colon cancer patients in [102,129]. The study in [102] also shows that a high level of regulatory T-cells is related to poor prognosis of patients, which supports our results that regulatory T-cells decrease in density as cancer cells increase in density. Another similar finding between [102] and our study is that the density of dendritic cells increases along with tumor progression (Figure 5).

There is a significant body of research analyzing statistical and mathematical relations of particular components of tumor microenvironment and the disease progression and outcome for subsequent establishment of prognostic biomarkers [130,131,132,133,134,135,136,137,138,139]. Our result demonstrates that the dynamics of cancer development cannot be captured by one specific biomarker, but can rather be characterized by complex time-dependent interactions between many components of the immune system and tumor tissue. It is important to further develop and analyze these tumor–immune cell interactions and how they affect different possibilities of treatment.

It is important to point out that, similar to many other mathematical models of biological processes, this model has some limitations that arise from the lack of time course data sets. Specifically, we make the assumption that the largest tumors in each cluster are the evolution of the smallest ones. Importantly, as we mentioned above, some of the predictions of the model match with biological observations. However, these facts do not provide a complete validation for the model, and the model should be validated on a separate time course data. We hope that this work encourages scientists to collect time course data. Although this work has some limitations, it provides important insights and an opportunity for scientists to improve the mathematical model of colonic tumors and/or validate the model if they have more data or insights.

One way forward is the design of patient-specific models [140,141,142,143]. These models can utilize the tumor immune microenvironment deconvolution and clustering methods for available patient data as detailed in this paper. New prognosis can be built based on established dynamics from patients with similar immune characteristics. To better match the dynamics of the model to real patient data, various parameter fitting algorithms can be utilized [144,145,146,147]. Another possible improvement is a transition to a partial differential equations model [148] to analyze spatial properties of tumor development as well as temporal.

## Figures and Tables

**Table 1:** **Model’s variables.** Names and descriptions of variables used in the model.

Variable	Name	Description
$T_{N}$	Naive T-cells	
$T_{h}$	Helper T-cells	
$T_{C}$	Cytotoxic cells	includes CD8+ T-cells and NK cells
$T_{r}$	Regulatory T-cells	
$D_{n}$	Naive dendritic cells	
*D*	Activated dendritic cells	antigen presenting cells
*M*	Macrophages	
*C*	Cancer cells	
*N*	Nectrotic cells	
*H*	HMGB1	
$\mu_{1}$	Carcinogenic cytokines	includes effects of IL-6, IL-17, IL-21 and IL-22
$\mu_{2}$	Immunosuppresive agents	includes effects of IL-10 and CCL20
$I_{\gamma }$	IFN-γ	
$G_{\beta }$	TGF-β	

**Table 2:** **Steady-state cell densities.** We group large tumors in each cluster and calculate their average cell densities in cells/cm^3^. These data are used for parameter derivation detailed in Appendix B.

Cluster	$T_{N}^{\infty }$	$T_{h}^{\infty }$	$T_{C}^{\infty }$	$T_{r}^{\infty }$	$D_{N}^{\infty }$	$D^{\infty }$	$M^{\infty }$	$M_{0}$
1	1.4914 $\times \;10^{4}$	4.6358 $\times \;10^{3}$	2.5845 $\times \;10^{3}$	2.3891 $\times \;10^{3}$	3.0504 $\times \;10^{2}$	6.0214 $\times \;10^{2}$	1.1798 $\times \;10^{4}$	2.1004 $\times \;10^{4}$
2	1.1429 $\times \;10^{4}$	6.0411 $\times \;10^{3}$	5.3853 $\times \;10^{3}$	3.3646 $\times \;10^{3}$	1.0329 $\times \;10^{2}$	5.1299 $\times \;10^{2}$	8.6227 $\times \;10^{3}$	1.6445 $\times \;10^{4}$
3	9.2381 $\times \;10^{3}$	1.3864 $\times \;10^{3}$	1.1139 $\times \;10^{3}$	2.7910 $\times \;10^{3}$	1.8878 $\times \;10^{-1}$	1.8635 $\times \;10^{2}$	6.7972 $\times \;10^{3}$	3.2146 $\times \;10^{4}$
4	1.3878 $\times \;10^{4}$	2.4910 $\times \;10^{3}$	3.2172 $\times \;10^{3}$	2.2783 $\times \;10^{3}$	1.4196 $\times \;10^{2}$	6.2154 $\times \;10^{2}$	1.2931 $\times \;10^{4}$	1.5761 $\times \;10^{4}$
5	1.0262 $\times \;10^{4}$	3.7844 $\times \;10^{3}$	1.6853 $\times \;10^{3}$	2.6394 $\times \;10^{3}$	8.0199 ×10	1.9084 $\times \;10^{2}$	1.1603 $\times \;10^{4}$	2.8198 $\times \;10^{4}$
	$C^{\infty }$	$N^{\infty }$	$\mu_{1}^{\infty }$	$\mu_{2}^{\infty }$	$H^{\infty }$	$I_{\gamma }^{\infty }$	$G_{\beta }^{\infty }$	
1	9.1531 $\times \;10^{4}$	4.5765 $\times \;10^{4}$	1.6328 $\times \;10^{2}$	1.2987 $\times \;10^{3}$	8.9811 $\times \;10^{3}$	8.5737	1.9037 $\times \;10^{4}$	
2	9.7064 $\times \;10^{4}$	4.8532 $\times \;10^{4}$	1.7552 $\times \;10^{2}$	1.3249 $\times \;10^{3}$	8.5279 $\times \;10^{3}$	10.5677	2.2275 $\times \;10^{4}$	
3	9.0029 $\times \;10^{4}$	4.5014 $\times \;10^{4}$	1.9866 $\times \;10^{2}$	1.2906 $\times \;10^{3}$	9.5122 $\times \;10^{3}$	0.8287	2.5145 $\times \;10^{4}$	
4	9.6956 $\times \;10^{4}$	4.8478 $\times \;10^{4}$	1.1410 $\times \;10^{2}$	3.3689 $\times \;10^{2}$	5.1782 $\times \;10^{3}$	1.2703	8.1734 $\times \;10^{3}$	
5	8.0584 $\times \;10^{4}$	4.0292 $\times \;10^{4}$	1.2058 $\times \;10^{2}$	7.1551 $\times \;10^{2}$	7.7848 $\times \;10^{3}$	5.7892	2.6260 $\times \;10^{4}$	

**Table 3:** **Dimensionless initial conditions.** Values of initial conditions for the dimensionless system derived from the patients with the smallest tumor size.

Cluster	$T_{N}/T_{N}^{\infty }$	$T_{h}/T_{h}^{\infty }$	$T_{C}/T_{C}^{\infty }$	$T_{r}/T_{r}^{\infty }$	$D_{N}/D_{N}^{\infty }$	$D/D^{\infty }$	$M/M^{\infty }$
1	0.9311	1.2492	2.4626	0.6872	1.6328	0.0003	0.6737
2	1.2302	1.3155	1.5210	0.5107	2.0461	2.7822	1.2920
3	1.1997	0.8555	1.6948 $\times \;10^{-4}$	0.6572	1.0000	1.0130 $\times \;10^{-3}$	1.4150
4	1.4471	0.1571	0.5823	0.8910	5.6827	4.2945	0.9259
5	0.6794	2.6119	1.6294	1.8819	2.3538 $\times \;10^{-3}$	0.4542	0.7749
	$C/C^{\infty }$	$N/N^{\infty }$	$\mu_{1}/\mu_{1}^{\infty }$	$\mu_{2}/\mu_{2}^{\infty }$	$H/H^{\infty }$	$I_{\gamma }/I_{\gamma }^{\infty }$	$G_{\beta }/G_{\beta }^{\infty }$
1	3.1466 $\times \;10^{-4}$	0.0	0.4971	0.5124	1.4712	3.8892	0.2549
2	2.9672 $\times \;10^{-4}$	0.0	0.7578	0.1790	0.6036	0.9385	0.5566
3	3.1991 $\times \;10^{-4}$	0.0	0.1335	0.8419	1.2566	0.0	0.6851
4	2.9706 $\times \;10^{-4}$	0.0	0.4137	5.7720	1.4630	0.0	2.5629
5	3.5741 $\times \;10^{-4}$	0.0	0.4587	2.2979	1.1835	0.4084	0.3457

## Data Availability

Publicly available TCGA clinical data was downloaded from GDC portal. Figure 2 was obtained using TumorDecon software: https://github.com/ShahriyariLab/TumorDecon. Python scripts for computations and MatLab scripts for plotting the results presented on Figure 4, Figure 5 and Figure 6 and Figure A2, Figure A3, Figure A4, Figure A5, Figure A6 are available here: https://github.com/ShahriyariLab/Data-driven-mathematical-model-for-colon-cancer.

## Abbreviations

The following abbreviations are used in this manuscript:

CAC	colitis-associated cancer
CCL20	chemokine (C-C motif) ligand 20
COAD	colon adenocarcinoma
DAMP	damage-associated molecular pattern
DCs	dendritic cells
FasL	fas ligand
GDC	Genomic Data Commons
GEP	gene expression profiles
HMGB1	high mobility group box 1
IFN	interferon
IL	interleukin
NF-κB	nuclear factor kappa B
NK cells	natural killer cells
ODE	ordinary differential equation
RAGE	receptor for advanced glycation endproducts
RNA-seq	ribonucleic acid sequencing
STAT	signal transducer and activator of transcription
TAM	tumor associated macrophage
TCGA	the cancer genome atlas
TGF	transforming growth factor
TNF	tumor necrosis factor
TSLP	thymic stromal lymphopoietin
UCSC	University of California Santa Cruz

## Appendix A. ODE System and Analysis

Combining Equations (1)–(14), we obtain the following system

$$\begin{array}{cc} \frac{d\left[T_{N}\right]}{dt}= & A_{T_{N}}-\left(\lambda_{T_{h}D}\left[D\right]+\lambda_{T_{h}M}\left[M\right]+\lambda_{T_{h}\mu_{1}}\left[\mu_{1}\right]\right)\left[T_{N}\right]-\left(\lambda_{T_{C}T_{h}}\left[T_{h}\right]+\lambda_{T_{C}D}\left[D\right]\right)\left[T_{N}\right] \end{array}$$

(A1) $$\begin{array}{cc} \; & -\left(\lambda_{T_{r}T_{h}}\left[T_{h}\right]+\lambda_{T_{r}\mu_{2}}\left[\mu_{2}\right]+\lambda_{T_{r}G_{\beta }}\left[G_{\beta }\right]\right)\left[T_{N}\right]-\delta_{T_{N}}\left[T_{N}\right] \end{array}$$

(A2) $$\begin{array}{cc} \frac{d\left[T_{h}\right]}{dt}= & \left(\lambda_{T_{h}D}\left[D\right]+\lambda_{T_{h}M}\left[M\right]+\lambda_{T_{h}\mu_{1}}\left[\mu_{1}\right]\right)\left[T_{N}\right]-\left(\delta_{T_{h}\mu_{2}}\left[\mu_{2}\right]+\delta_{T_{h}T_{r}}\left[T_{r}\right]+\delta_{T_{h}}\right)\left[T_{h}\right] \end{array}$$

(A3) $$\begin{array}{cc} \frac{d\left[T_{C}\right]}{dt}= & \left(\lambda_{T_{C}T_{h}}\left[T_{h}\right]+\lambda_{T_{C}D}\left[D\right]\right)\left[T_{N}\right]-\left(\delta_{T_{C}\mu_{2}}\left[\mu_{2}\right]+\delta_{T_{C}T_{r}}\left[T_{r}\right]+\delta_{T_{C}}\right)\left[T_{C}\right] \end{array}$$

(A4) $$\begin{array}{cc} \frac{d\left[T_{r}\right]}{dt}= & \left(\lambda_{T_{r}T_{h}}\left[T_{h}\right]+\lambda_{T_{r}\mu_{2}}\left[\mu_{2}\right]+\lambda_{T_{r}G_{\beta }}\left[G_{\beta }\right]\right)\left[T_{N}\right]-\left(\delta_{T_{r}\mu_{1}}\left[\mu_{1}\right]+\delta_{T_{r}}\right)\left[T_{r}\right] \end{array}$$

(A5) $$\begin{array}{cc} \frac{d\left[D_{N}\right]}{dt}= & A_{D_{N}}-\left(\lambda_{DH}\left[H\right]+\lambda_{DC}\left[C\right]\right)\left[D_{N}\right]-\left(\delta_{DH}\left[H\right]+\delta_{D}\right)\left[D_{N}\right] \end{array}$$

(A6) $$\begin{array}{cc} \frac{d\left[D\right]}{dt}= & \left(\lambda_{DH}\left[H\right]+\lambda_{DC}\left[C\right]\right)\left[D_{N}\right]-\left(\delta_{DH}\left[H\right]+\delta_{DC}\left[C\right]+\delta_{D}\right)\left[D\right] \end{array}$$

(A7) $$\begin{array}{cc} \frac{d\left[M\right]}{dt}= & \left(\lambda_{M\mu_{2}}\left[\mu_{2}\right]+\lambda_{MI_{\gamma }}\left[I_{\gamma }\right]+\lambda_{MT_{h}}\left[T_{h}\right]\right)\left(M_{0}-\left[M\right]\right)-\delta_{M}\left[M\right] \end{array}$$

(A8) $$\begin{array}{cc} \frac{d\left[C\right]}{dt}= & \left(\lambda_{C}+\lambda_{C\mu_{1}}\left[\mu_{1}\right]\right)\left[C\right]\left(1-\frac{\left[C\right]}{C_{0}}\right)-\left(\delta_{CG_{\beta }}\left[G_{\beta }\right]+\delta_{CI_{\gamma }}\left[I_{\gamma }\right]+\delta_{CT_{C}}\left[T_{C}\right]+\delta_{C}\right)\left[C\right] \end{array}$$

(A9) $$\begin{array}{cc} \frac{d\left[N\right]}{dt}= & \alpha_{NC}\left(\delta_{CG_{\beta }}\left[G_{\beta }\right]+\delta_{CI_{\gamma }}\left[I_{\gamma }\right]+\delta_{CT_{C}}\left[T_{C}\right]+\delta_{C}\right)\left[C\right]-\delta_{N}\left[N\right] \end{array}$$

(A10) $$\begin{array}{cc} \frac{d\left[H\right]}{dt}= & \lambda_{HN}\left[N\right]+\lambda_{HM}\left[M\right]+\lambda_{HT_{h}}\left[T_{h}\right]+\lambda_{HT_{C}}\left[T_{C}\right]+\lambda_{HT_{r}}\left[T_{r}\right]-\delta_{H}\left[H\right] \end{array}$$

(A11) $$\begin{array}{cc} \frac{d\left[\mu_{1}\right]}{dt}= & \lambda_{\mu_{1}T_{h}}\left[T_{h}\right]+\lambda_{\mu_{1}M}\left[M\right]+\lambda_{\mu_{1}D}\left[D\right]-\delta_{\mu_{1}}\left[\mu_{1}\right] \end{array}$$

(A12) $$\begin{array}{cc} \frac{d\left[\mu_{2}\right]}{dt}= & \lambda_{\mu_{2}M}\left[M\right]+\lambda_{\mu_{2}D}\left[D\right]+\lambda_{\mu_{2}T_{r}}\left[T_{r}\right]-\delta_{\mu_{2}}\left[\mu_{2}\right] \end{array}$$

(A13) $$\begin{array}{cc} \frac{d\left[I_{\gamma }\right]}{dt}= & \lambda_{I_{\gamma }T_{h}}\left[T_{h}\right]+\lambda_{I_{\gamma }T_{C}}\left[T_{C}\right]+\lambda_{I_{\gamma }M}\left[M\right]-\delta_{I_{\gamma }}\left[I_{\gamma }\right] \end{array}$$

(A14) $$\begin{array}{cc} \frac{d\left[G_{\beta }\right]}{dt}= & \lambda_{G_{\beta }M}\left[M\right]+\lambda_{G_{\beta }T_{r}}\left[T_{r}\right]-\delta_{G_{\beta }}\left[G_{\beta }\right] \end{array}$$

The system has 14 variables and 59 different parameters. The λ parameters correspond to proliferation, activation and production rates, δ parameters correspond to degradation and cell death rates and four parameters: $A_{T_{N}}$ and $A_{D_{N}}$ respectively are the production rates of naive T-cells and dendritic cells, $M_{0}$ and $C_{0}$ are the total density of macrophages (naive and activated together) and cancer cells maximum capacity, respectively.

### Appendix A.1. Positivity

To prove that the system with positive coefficients and positive initial conditions has positive solution, let us consider the set of integrating factors, one for each variable:

$$\begin{array}{cc} \eta_{T_{N}}\left(t\right)= & \exp\int_{0}^{t}\left(\left(\lambda_{T_{h}D}+\lambda_{T_{c}D}\right)\left[D\right]+\lambda_{T_{h}M}\left[M\right]+\lambda_{T_{h}\mu_{1}}\left[\mu_{1}\right]+\right.\\ \; & \;\left.+\left(\lambda_{T_{c}T_{h}}+\lambda_{T_{r}T_{h}}\right)\left[T_{h}\right]+\lambda_{T_{r}\mu_{2}}\left[\mu_{2}\right]+\lambda_{T_{r}G_{\beta }}\left[G_{\beta }\right]+\delta_{T_{N}}\right)ds,\\ \eta_{T_{h}}\left(t\right)= & \exp\int_{0}^{t}\left(\delta_{T_{h}\mu_{2}}\left[\mu_{2}\right]+\delta_{T_{h}T_{r}}\left[T_{r}\right]+\delta_{T_{h}}\right)ds,\\ \eta_{T_{C}}\left(t\right)= & \exp\int_{0}^{t}\left(\delta_{T_{C}\mu_{2}}\left[\mu_{2}\right]+\delta_{T_{C}T_{r}}\left[T_{r}\right]+\delta_{T_{C}}\right)ds,\\ \eta_{T_{r}}\left(t\right)= & \exp\int_{0}^{t}\left(\delta_{T_{r}\mu_{1}}\left[\mu_{1}\right]+\delta_{T_{r}}\right)ds,\\ \eta_{D_{N}}\left(t\right)= & \exp\int_{0}^{t}\left(\lambda_{DH}\left[H\right]+\lambda_{DC}\left[C\right]+\delta_{DH}\left[H\right]+\delta_{D}\right)ds,\\ \eta_{D}\left(t\right)= & \exp\int_{0}^{t}\left(\delta_{DH}\left[H\right]+\delta_{DC}\left[C\right]+\delta_{D}\right)ds,\\ \eta_{M}\left(t\right)= & \exp\int_{0}^{t}\left(\lambda_{M\mu_{2}}\left[\mu_{2}\right]+\lambda_{MI_{\gamma }}\left[I_{\gamma }\right]+\lambda_{MT_{h}}\left[T_{h}\right]+\delta_{M}\right)ds,\\ \eta_{C}\left(t\right)= & \exp\int_{0}^{t}\left(\delta_{CG_{\beta }}\left[G_{\beta }\right]+\delta_{CI_{\gamma }}\left[I_{\gamma }\right]+\delta_{CT_{C}}\left[T_{C}\right]+\delta_{C}-\left(\lambda_{C}+\lambda_{C\mu_{1}}\left[\mu_{1}\right]\right)\left(1-\frac{\left[C\right]}{C_{0}}\right)\right)ds,\\ \eta_{N}\left(t\right)= & \exp\left(\delta_{N}t\right),\;\eta_{H}\left(t\right)=\exp\left(\delta_{H}t\right),\;\eta_{\mu_{1}}\left(t\right)=\exp\left(\delta_{\mu_{1}}t\right),\\ \eta_{\mu_{2}}\left(t\right)= & \exp\left(\delta_{\mu_{2}}t\right),\;\eta_{I_{\gamma }}\left(t\right)=\exp\left(\delta_{I_{\gamma }}t\right),\eta_{G_{\beta }}\left(t\right)=\exp\left(\delta_{G_{\beta }}t\right) \end{array}$$

These integrating factors will not allow us to derive explicit solution as some of them are defined through the unknown variables. It is important to note that the factors are strictly positive and allow us to rewrite the system as

$$\begin{array}{cc} \frac{d\left(\left[T_{N}\right]\eta_{T_{N}}\right)}{dt}= & A_{T_{N}}\eta_{T_{N}}\\ \frac{d\left(\left[T_{h}\right]\eta_{T_{h}}\right)}{dt}= & \left(\lambda_{T_{h}D}\left[D\right]+\lambda_{T_{h}M}\left[M\right]+\lambda_{T_{h}\mu_{1}}\left[\mu_{1}\right]\right)\left[T_{N}\right]\eta_{T_{h}}\\ \frac{d\left(\left[T_{C}\right]\eta_{T_{C}}\right)}{dt}= & \left(\lambda_{T_{C}T_{h}}\left[T_{h}\right]+\lambda_{T_{C}D}\left[D\right]\right)\left[T_{N}\right]\eta_{T_{C}}\\ \frac{d\left(\left[T_{r}\right]\eta_{T_{r}}\right)}{dt}= & \left(\lambda_{T_{r}T_{h}}\left[T_{h}\right]+\lambda_{T_{r}\mu_{2}}\left[\mu_{2}\right]+\lambda_{T_{r}G_{\beta }}\left[G_{\beta }\right]\right)\left[T_{N}\right]\eta_{T_{r}}\\ \frac{d\left(\left[D_{N}\right]\eta_{D_{N}}\right)}{dt}= & A_{D_{N}}\eta_{D_{N}}\\ \frac{d\left(\left[D\right]\eta_{D}\right)}{dt}= & \left(\lambda_{DH}\left[H\right]+\lambda_{DC}\left[C\right]\right)\left[D_{N}\right]\eta_{D}\\ \frac{d\left(\left[M\right]\eta_{M}\right)}{dt}= & \left(\lambda_{M\mu_{2}}\left[\mu_{2}\right]+\lambda_{MI_{\gamma }}\left[I_{\gamma }\right]+\lambda_{MT_{h}}\left[T_{h}\right]\right)M_{0}\eta_{M}\\ \frac{d\left(\left[C\right]\eta_{C}\right)}{dt}= & 0\\ \frac{d\left(\left[N\right]\eta_{N}\right)}{dt}= & \alpha_{NC}\left(\delta_{CG_{\beta }}\left[G_{\beta }\right]+\delta_{CI_{\gamma }}\left[I_{\gamma }\right]+\delta_{CT_{C}}\left[T_{C}\right]+\delta_{C}\right)\left[C\right]\eta_{N}\\ \frac{d\left(\left[H\right]\eta_{H}\right)}{dt}= & \left(\lambda_{HN}\left[N\right]+\lambda_{HM}\left[M\right]+\lambda_{HT_{h}}\left[T_{h}\right]+\lambda_{HT_{C}}\left[T_{C}\right]+\lambda_{HT_{r}}\left[T_{r}\right]\right)\eta_{H}\\ \frac{d\left(\left[\mu_{1}\right]\eta_{\mu_{1}}\right)}{dt}= & \left(\lambda_{\mu_{1}T_{h}}\left[T_{h}\right]+\lambda_{\mu_{1}M}\left[M\right]+\lambda_{\mu_{1}D}\left[D\right]\right)\eta_{\mu_{1}}\\ \frac{d\left(\left[\mu_{2}\right]\eta_{\mu_{2}}\right)}{dt}= & \left(\lambda_{\mu_{2}M}\left[M\right]+\lambda_{\mu_{2}D}\left[D\right]+\lambda_{\mu_{2}T_{r}}\left[T_{r}\right]\right)\eta_{\mu_{2}}\\ \frac{d\left(\left[I_{\gamma }\right]\eta_{I_{\gamma }}\right)}{dt}= & \left(\lambda_{I_{\gamma }T_{h}}\left[T_{h}\right]+\lambda_{I_{\gamma }T_{C}}\left[T_{C}\right]+\lambda_{I_{\gamma }M}\left[M\right]\right)\eta_{I_{\gamma }}\\ \frac{d\left(\left[G_{\beta }\right]\eta_{G_{\beta }}\right)}{dt}= & \left(\lambda_{G_{\beta }M}\left[M\right]+\lambda_{G_{\beta }T_{r}}\left[T_{r}\right]\right)\eta_{G_{\beta }} \end{array}$$

We see that the right-hand side of each equation in this system is non-negative, which means that the variable-factor product is non-decreasing, and thus if positive initially remains positive at all times.

### Appendix A.2. Boundedness

Let us show that all positive solutions are bounded for positive time *t*. We split the equations into groups by cell types. It is important to note, that we are not trying to derive the sharp bounds, just show that the bounds exist.

#### Appendix A.2.1. T-Cells

Adding Equations (A1)–(A4), we get

$$\begin{array}{cc} \frac{d\left(\left[T_{N}\right]+\left[T_{h}\right]+\left[T_{C}\right]+\left[T_{r}\right]\right)}{dt}= & A_{T_{N}}-\delta_{T_{N}}\left[T_{N}\right]-\left(\delta_{T_{h}\mu_{2}}\left[\mu_{2}\right]+\delta_{T_{h}T_{r}}\left[T_{r}\right]+\delta_{T_{h}}\right)\left[T_{h}\right]\\ \; & -\left(\delta_{T_{C}\mu_{2}}\left[\mu_{2}\right]+\delta_{T_{C}T_{r}}\left[T_{r}\right]+\delta_{T_{C}}\right)\left[T_{C}\right]-\left(\delta_{T_{r}\mu_{1}}\left[\mu_{1}\right]+\delta_{T_{r}}\right)\left[T_{r}\right]\\ \leq & A_{T_{N}}-\left(\left[T_{N}\right]+\left[T_{h}\right]+\left[T_{C}\right]+\left[T_{r}\right]\right)\min\left(\delta_{T_{N}},\delta_{T_{h}},\delta_{T_{C}},\delta_{T_{r}}\right). \end{array}$$

Integrating this inequality, we obtain

$$\begin{array}{cc} \left(\left[T_{N}\right]+\left[T_{h}\right]+\left[T_{C}\right]+\left[T_{r}\right]\right)\leq & \frac{A_{T_{N}}}{\min\left(\delta_{T_{N}},\delta_{T_{h}},\delta_{T_{C}},\delta_{T_{r}}\right)}\left(1-e^{-\min\left(\delta_{T_{N}},\delta_{T_{h}},\delta_{T_{C}},\delta_{T_{r}}\right)t}\right)\\ \; & +\left(\left[T_{N}\right]\left(0\right)+\left[T_{h}\right]\left(0\right)+\left[T_{C}\right]\left(0\right)+\left[T_{r}\right]\left(0\right)\right)e^{-\min\left(\delta_{T_{N}},\delta_{T_{h}},\delta_{T_{C}},\delta_{T_{r}}\right)t}. \end{array}$$

The right-hand side function is bounded, and since we have proven that each cell density is positive, all T-cells have to remain bounded.

#### Appendix A.2.2. Dendritic Cells

Let us add Equations (A5) and (A6) to obtain

$$\frac{d\left(\left[D_{N}\right]+\left[D\right]\right)}{dt}=A_{D_{N}}-\left(\delta_{DH}\left[H\right]+\delta_{D}\right)\left(\left[D_{N}\right]+\left[D\right]\right)-\delta_{DC}\left[C\right]\left[D\right]\leq A_{D_{N}}-\delta_{D}\left(\left[D_{N}\right]+\left[D\right]\right).$$

Integrating, we get

$$\left(\left[D_{N}\right]+\left[D\right]\right)\leq \frac{A_{D_{N}}}{\delta_{D}}\left(1-e^{-\delta_{D}t}\right)+\left(\left[D_{N}\right]\left(0\right)+\left[D\right]\left(0\right)\right)e^{-\delta_{D}t}.$$

Since the right-hand side is bounded and each variable is positive, this proves that each variable is bounded.

#### Appendix A.2.3. Macrophages

Let us rewrite Equation (A7) as

$$\frac{d\left(\left[M\right]-M_{0}\right)}{dt}-\left(\lambda_{M\mu_{2}}\left[\mu_{2}\right]+\lambda_{MI_{\gamma }}\left[I_{\gamma }\right]+\lambda_{MT_{h}}\left[T_{h}\right]\right)\left(M_{0}-\left[M\right]\right)=-\delta_{M}\left[M\right]\leq 0.$$

Integrating (with implicit dependence on variables $\left[\mu_{2}\right]$, $\left[I_{\gamma }\right]$, and $\left[T_{h}\right]$) results in

$$\left[M\right]\leq M_{0}-\left(M_{0}-\left[M\right]\left(0\right)\right)\exp\left(-\int_{0}^{t}\left(\lambda_{M\mu_{2}}\left[\mu_{2}\right]\left(s\right)+\lambda_{MI_{\gamma }}\left[I_{\gamma }\right]\left(s\right)+\lambda_{MT_{h}}\left[T_{h}\right]\left(s\right)\right)ds\right).$$

The right-hand side function is bounded for positive $\left[\mu_{2}\right]$, $\left[I_{\gamma }\right]$ and $\left[T_{h}\right]$, and thus proves the bound on $\left[M\right]$.

#### Appendix A.2.4. Cancer Cells

Rewriting the Equation (A8) as

$$\frac{d\left(\left[C\right]-C_{0}\right)}{dt}-\frac{\left(\lambda_{C}+\lambda_{C\mu_{1}}\left[\mu_{1}\right]\right)\left[C\right]}{C_{0}}\left(C_{0}-\left[C\right]\right)=\left(\delta_{CG_{\beta }}\left[G_{\beta }\right]+\delta_{CI_{\gamma }}\left[I_{\gamma }\right]+\delta_{CT_{C}}\left[T_{C}\right]+\delta_{C}\right)\left[C\right]\leq 0$$

we integrate with implicit dependence on both $\left[C\right]$ and $\left[\mu_{1}\right]$ to obtain

$$\left[C\right]\leq C_{0}-\left(C_{0}-\left[C\right]\left(0\right)\right)\exp\left(-\int_{0}^{t}\frac{\left(\lambda_{C}+\lambda_{C\mu_{1}}\left[\mu_{1}\right]\left(s\right)\right)\left[C\right]\left(s\right)}{C_{0}}ds\right).$$

Since $\left[C\right]$ and $\left[\mu_{1}\right]$ are proven to remain positive, the right-hand side is bounded, hence $\left[C\right]$ is bounded.

#### Appendix A.2.5. Interferon-γ and TGF-β

Here we show the bound on $\left[I_{\gamma }\right]$ and $\left[G_{\beta }\right]$ as we need them to prove the bound on $\left[N\right]$.

**Remark** **A1.** *Alternatively we could show a bound on*$\left[\mu_{1}\right]$*and subsequent bound on*$\left[N\right]+\left[C\right]$.
*Observe that on the right-hand sides of Equations (A13) and (A14), the positive terms are already proven to be bounded:*

$$\begin{array}{cc} \lambda_{I_{\gamma }T_{h}}\left[T_{h}\right]+\lambda_{I_{\gamma }T_{C}}\left[T_{C}\right]+\lambda_{I_{\gamma }M}\left[M\right] & \leq \lambda_{I_{\gamma }}^{max},\\ \lambda_{G_{\beta }M}\left[M\right]+\lambda_{G_{\beta }T_{r}}\left[T_{r}\right] & \leq \lambda_{G_{\beta }}^{max}. \end{array}$$

*Then combining these with Equations (A13) and (A14) we get the following differential inequalities:*

$$\frac{d\left[I_{\gamma }\right]}{dt}\leq \lambda_{I_{\gamma }}^{max}-\delta_{I_{\gamma }}\left[I_{\gamma }\right],\;\frac{d\left[G_{\beta }\right]}{dt}\leq \lambda_{G_{\beta }}^{max}-\delta_{G_{\beta }}\left[G_{\beta }\right].$$

*Integrating, we get*

$$\begin{array}{cc} \left[I_{\gamma }\right]\leq & \frac{\lambda_{I_{\gamma }}^{max}}{\delta_{I_{\gamma }}}\left(1-e^{-\delta_{I_{\gamma }}t}\right)+\left[I_{\gamma }\right]\left(0\right)e^{-\delta_{I_{\gamma }}t},\\ \left[G_{\beta }\right]\leq & \frac{\lambda_{G_{\beta }}^{max}}{\delta_{G_{\beta }}}\left(1-e^{-\delta_{G_{\beta }}t}\right)+\left[G_{\beta }\right]\left(0\right)e^{-\delta_{G_{\beta }}t}, \end{array}$$

*which proves the bound.*

#### Appendix A.2.6. Necrotic Cells

Now we notice that for Equation (A9) all the components of the positive term are already proven to remain bounded

$$\alpha_{NC}\left(\delta_{CG_{\beta }}\left[G_{\beta }\right]+\delta_{CI_{\gamma }}\left[I_{\gamma }\right]+\delta_{CT_{C}}\left[T_{C}\right]+\delta_{C}\right)\left[C\right]\leq \lambda_{N}^{max},$$

which results in the differential inequality

$$\frac{d\left[N\right]}{dt}\leq \lambda_{N}^{max}-\delta_{N}\left[N\right],$$

subsequently resulting after integration in the following bound:

$$\left[N\right]\leq \frac{\lambda_{N}^{max}}{\delta_{N}}\left(1-e^{-\delta_{N}t}\right)+\left[N\right]\left(0\right)e^{-\delta_{N}t}.$$

#### Appendix A.2.7. Remaining Cytokines

With the bound on necrotic cells we have proven boundedness for all the positive components of the right-hand sides of the Equations (A10)–(A12):

$$\begin{array}{cc} \lambda_{HN}\left[N\right]+\lambda_{HM}\left[M\right]+\lambda_{HT_{h}}\left[T_{h}\right]+\lambda_{HT_{C}}\left[T_{C}\right]+\lambda_{HT_{r}}\left[T_{r}\right] & \leq \lambda_{H}^{max},\\ \lambda_{\mu_{1}T_{h}}\left[T_{h}\right]+\lambda_{\mu_{1}M}\left[M\right]+\lambda_{\mu_{1}D}\left[D\right] & \leq \lambda_{\mu_{1}}^{max},\\ \lambda_{\mu_{2}M}\left[M\right]+\lambda_{\mu_{2}D}\left[D\right]+\lambda_{\mu_{2}T_{r}}\left[T_{r}\right] & \leq \lambda_{\mu_{2}}^{max}. \end{array}$$

Thus, the following differential inequalities are valid:

$$\begin{array}{cc} \frac{d\left[H\right]}{dt}\leq & \;\lambda_{H}^{max}-\delta_{H}\left[H\right],\\ \frac{d\left[\mu_{1}\right]}{dt}\leq & \;\lambda_{\mu_{1}}^{max}-\delta_{\mu_{1}}\left[\mu_{1}\right],\\ \frac{d\left[\mu_{2}\right]}{dt}\leq & \;\lambda_{\mu_{2}}^{max}-\delta_{\mu_{2}}\left[\mu_{2}\right]. \end{array}$$

Integrating, we obtain

$$\begin{array}{cc} \left[H\right]\leq & \;\frac{\lambda_{H}^{max}}{\delta_{H}}\left(1-e^{-\delta_{H}t}\right)+\left[H\right]\left(0\right)e^{-\delta_{H}t},\\ \left[\mu_{1}\right]\leq & \;\frac{\lambda_{\mu_{1}}^{max}}{\delta_{\mu_{1}}}\left(1-e^{-\delta_{\mu_{1}}t}\right)+\left[\mu_{1}\right]\left(0\right)e^{-\delta_{\mu_{1}}t},\\ \left[\mu_{2}\right]\leq & \;\frac{\lambda_{\mu_{2}}^{max}}{\delta_{\mu_{2}}}\left(1-e^{-\delta_{\mu_{2}}t}\right)+\left[\mu_{2}\right]\left(0\right)e^{-\delta_{\mu_{2}}t}. \end{array}$$

Thus, $\left[H\right]$, $\left[\mu_{1}\right]$ and $\left[\mu_{2}\right]$ are bounded for positive *t*.

## Appendix B. Derivation of the Sample Parameter Set

### Appendix B.1. Steady-State and Additional Assumptions

We derive the sample parameter set under the assumption of specific values of steady-state for each variable:

$$T_{N}^{\infty },\;T_{h}^{\infty },\;T_{C}^{\infty },\;T_{r}^{\infty },\;D_{N}^{\infty },\;D^{\infty },\;M^{\infty },\;C^{\infty },\;N^{\infty },\;H^{\infty },\;\mu_{1}^{\infty },\;\mu_{2}^{\infty },\;I_{\gamma }^{\infty },\;G_{\beta }^{\infty }.$$

Then, Equations (A1)–(A14) provide us with 14 restrictions on parameters. Since the number of unknown parameters is larger than the number of equations at the steady-state, we make further assumptions about some of parameters in order to have the same number of independent equations as the number of unknown parameters and to be able to uniquely determine the values. We assume, given cancer cell and macrophage capacities, $C_{0}$ and $M_{0}$, as well as necrosis coefficient $\alpha_{NC}=3/4$. Additionally, from the available research [126,130,149,150,151,152,153,154,155,156,157] we adopt the natural decay/death/degradation rates. For some of the specimen, considering a specimen *X*, we will estimate death rate $\delta_{X}$ using published measurements of half-life $t_{1/2}^{X}$ using the following formula $\delta_{X}=\ln2/t_{1/2}^{X}$. Let us look at these in detail. According to [154], the half life of naive T-cells and memory T-cells range between 1–8 years and 1–12 months, respectively. We take the half-life of $T_{N}$ to be 2 years, respectively to $\delta_{T_{N}}=9.4951\times 10^{-4}$. Half-lives of CD8+ and CD4+ T-cells are estimated at 41 h and 3 days correspondingly [150]. This results in $\delta_{T_{h}}=\delta_{T_{r}}=0.231$ and $\delta_{T_{C}}=0.406$. Ref. [149] estimates half life of mature DC to be 2–3 days. Taking 2.5 days, we obtain $\delta_{D}=0.277$. The half-life of intestinal macrophages is approximately 4–6 weeks [158]. We consider a value $\delta_{M}=0.02$ from supplementary information of [151], closely corresponding to 5-week half-life. For $\mu_{1}$ we assume 15.5 h half-life of IL-6 [153], resulting in $\delta_{\mu_{1}}=1.07$. For $\mu_{2}$, we consider 2.7 to 4.5 h half-life of human IL-10 [155]. For the average 3.6 h the resulting decay rate is $\delta_{\mu_{2}}=4.62$. For HMGB1, we take half-life of 17 min [152] resulting in $\delta_{H}=58.7$. For IFN-γ, we assume half-life of 30 min [157] and corresponding rate $\delta_{I_{\gamma }}=33.27$. Lastly, for TGF-β, the estimated half-life is about 2 min [156] resulting in the rate $\delta_{G_{\beta }}=499$. To summarize, here are the death rates used in our computations:

(A15) $$\begin{array}{cccccccc} \delta_{T_{N}}= & 9.4951\times 10^{-4},\; & \delta_{T_{h}}= & 0.231,\; & \delta_{T_{C}}= & 0.406,\; & \delta_{T_{r}}= & 0.231, \end{array}$$

(A16) $$\begin{array}{cccccccc} \delta_{D}= & 0.277,\; & \delta_{M}= & 0.02,\; & \delta_{H}= & 58.7,\; & \delta_{I_{\gamma }}= & 33.27,\; \end{array}$$

(A17) $$\begin{array}{cccccc} \delta_{G_{\beta }}= & 499,\; & \delta_{\mu_{1}}= & 1.07,\; & \delta_{\mu_{2}}= & 4.62. \end{array}$$

As a last step, we impose heuristic assumptions on activation, inhibition and production rates. Let us look at these in detail.

First, we consider the results in [159] suggesting that range of colon cancer doubling time is between 92.4 and 1032.2 days. This range of doubling rates should include the effects of immune microenvironment. Thus, let us consider the doubling rate to be the difference between proliferation rate and death rate. Faster doubling rate includes both innate cancer proliferation and proliferation caused by $\mu_{1}$ family of cytokines, while death rate being only innate. This results in

(A18) $$\frac{\ln2}{92.4}\approx 7.5\times 10^{-3}=\left(\lambda_{C}+\lambda_{C\mu_{1}}\mu_{1}^{\mathrm{mean}}\right)-\delta_{C}.$$

On the other hand, for the slower doubling rate we only consider innate cancer proliferation, while death rate includes effects of all anti-cancer agents, i.e.,

(A19) $$\frac{\ln2}{1032.2}\approx 6.7152\times 10^{-4}=\lambda_{C}-\left(\delta_{CG_{\beta }}G_{\beta }^{\mathrm{mean}}+\delta_{CI_{\gamma }}I_{\gamma }^{\mathrm{mean}}+\delta_{CT_{C}}T_{C}^{\mathrm{mean}}+\delta_{C}\right).$$

Here we consider $\mu_{1}^{\mathrm{mean}},\;T_{C}^{\mathrm{mean}},\;I_{\gamma }^{\mathrm{mean}}$ and $G_{\beta }^{\mathrm{mean}}$ to be average values of the corresponding variable across all patients.

Further assumptions are based on maximal observable quantities for all the variable across all patients:

$$T_{N}^{\max},\;T_{h}^{\max},\;T_{C}^{\max},\;T_{r}^{\max},\;D_{N}^{\max},\;D^{\max},\;M^{\max},\;C^{\max},\;N^{\max},\;H^{\max},\;\mu_{1}^{\max},\;\mu_{2}^{\max},\;I_{\gamma }^{\max},\;G_{\beta }^{\max}.$$

See Appendix C for more details on patient data and specific values.

We assume that most of T-helper cells are activated by antigen-presenting dendritic cells, so we take

(A20) $$\lambda_{T_{h}D}D^{\max}=200\lambda_{T_{h}M}M^{\max}=200\lambda_{T_{h}\mu_{1}}\mu_{1}^{\max}.$$

We also assume that inhibition of T-helper cells by $\mu_{2}$ family of cytokines and by Treg cells are each 20 times more effective than the natural degradation:

(A21) $$\delta_{T_{h}\mu_{2}}\mu_{2}^{\max}=\delta_{T_{h}T_{r}}T_{r}^{\max}=20\delta_{T_{h}}.$$

For cytotoxic T-cells, we assume that activation by T-helper cells is twice as effective as activation by Dendritic cells

(A22) $$\lambda_{T_{C}T_{h}}T_{h}^{\max}=2\lambda_{T_{C}D}D^{\max},$$

and same as for T-helper cells inhibition of cytotoxic T-cells by $\mu_{2}$ family of cytokines and by Treg cells are each 20 times more effective than the natural degradation:

(A23) $$\delta_{T_{C}\mu_{2}}\mu_{2}^{\max}=\delta_{T_{C}T_{r}}T_{r}^{\max}=20\delta_{T_{C}}.$$

Next assumption is that activation of Treg cells by T-helper cells is four times larger than activation by $\mu_{2}$ family of cytokines and by TGF-β:

(A24) $$\lambda_{T_{r}T_{h}}T_{h}^{\max}=4\lambda_{T_{r}G_{\beta }}G_{\beta }^{\max},\;\lambda_{T_{r}\mu_{2}}\mu_{2}^{\max}=\lambda_{T_{r}G_{\beta }}G_{\beta }^{\max},$$

while inhibition of Treg cells by $\mu_{1}$ family of cytokines is 20 times larger than their natural degradation rate:

(A25) $$\delta_{T_{r}\mu_{1}}\mu_{1}^{\max}=20\delta_{T_{r}}.$$

We impose that activation of dendritic cells by HMGB1 is twice more effective than activation by cancer cells, inhibition of dendritic cells by HMGB1 is twice less effective than inhibiiton by cancer cells, and cumulative inhibition of dendritic cells by HMGB1 and cancer cells is equivalent to the natural degradation rate of dendritic cells:

(A26) $$\lambda_{DH}H^{\max}=2\lambda_{DC}C^{\max},\;\delta_{DH}H^{\max}=\frac{1}{2}\delta_{DC}C^{\max},\;\delta_{DH}H^{\max}+\delta_{DC}C^{\max}=\delta_{D}.$$

For macrophages, we assume that most macrophages are activated by T-helper cells, thus

(A27) $$\lambda_{MT_{h}}T_{h}^{\max}=10\lambda_{M\mu_{2}}\mu_{2}^{\max}=10\lambda_{MI_{\gamma }}I_{\gamma }^{\max}.$$

Next, we look at the cancer death rates. We assume TGF-β and IFN-γ are equally effective in killing cancer cells, but cytotoxic T-cells to be twice more effective, so

(A28) $$\delta_{CG_{\beta }}G_{\beta }^{\max}=\delta_{CI_{\gamma }}I_{\gamma }^{\max}=\frac{1}{2}\delta_{CT_{C}}T_{C}^{\max}.$$

We also assume that at its extreme value, TGF-β kills cancer cells 10 times faster than innate death rate of cancer cells:

(A29) $$\delta_{CG_{\beta }}G_{\beta }^{\max}=10\delta_{C}.$$

Next, let us list assumptions on production rates of cytokines per cell:

(A30) $$\begin{array}{cccccc} \lambda_{\mu_{1}M}= & 4\lambda_{\mu_{1}T_{h}}=8\lambda_{\mu_{1}D},\; & \lambda_{\mu_{2}D}= & \lambda_{\mu_{2}M}=\lambda_{\mu_{2}T_{r}},\; & \lambda_{HN}= & 10\lambda_{HM}=20\lambda_{HT_{h}},\\ \lambda_{HT_{h}}= & \lambda_{HT_{C}}=\lambda_{HT_{r}},\; & \lambda_{I_{\gamma }T_{c}}= & 4\lambda_{I_{\gamma }T_{h}}=20\lambda_{I_{\gamma }M},\; & \lambda_{G_{\beta }M}= & \lambda_{G_{\beta }T_{r}}. \end{array}$$

Altogether these assumptions are sufficient to derive a sample parameter set.

### Appendix B.2. Non-Dimensionalization

For more stable numerical simulations and to avoid scale dependence in the sensitivity analysis, we introduce non-dimensional variables. For each variable $\left[X\right]$ with steady-state $X^{\infty }$ we introduce non-dimensional $\left[\bar{X}\right]=\left[X\right]/X^{\infty }.$ Then for all the non-dimensional variables, steady-state will be equal to 1. As a result of the dramatic difference between timescales in different equations (related to natural decay rate), we make a choice to not scale time. Then we can rewrite the system as

(A31) $$\begin{array}{cc} \frac{d\left[{\bar{T}}_{N}\right]}{dt}= & {\bar{A}}_{T_{N}}-\alpha_{T_{N}T_{h}}\left({\bar{\lambda }}_{T_{h}D}\left[\bar{D}\right]+{\bar{\lambda }}_{T_{h}M}\left[\bar{M}\right]+{\bar{\lambda }}_{T_{h}\mu_{1}}\left[{\bar{\mu }}_{1}\right]\right)\left[{\bar{T}}_{N}\right]\\ \; & -\alpha_{T_{N}T_{C}}\left({\bar{\lambda }}_{T_{C}T_{h}}\left[{\bar{T}}_{h}\right]+{\bar{\lambda }}_{T_{C}D}\left[\bar{D}\right]\right)\left[{\bar{T}}_{N}\right]\\ \; & -\alpha_{T_{N}T_{r}}\left({\bar{\lambda }}_{T_{r}T_{h}}\left[{\bar{T}}_{h}\right]+{\bar{\lambda }}_{T_{r}\mu_{2}}\left[{\bar{\mu }}_{2}\right]+{\bar{\lambda }}_{T_{r}G_{\beta }}\left[{\bar{G}}_{\beta }\right]\right)\left[{\bar{T}}_{N}\right]-\delta_{T_{N}}\left[{\bar{T}}_{N}\right] \end{array}$$

(A32) $$\begin{array}{cc} \frac{d\left[{\bar{T}}_{h}\right]}{dt}= & \left({\bar{\lambda }}_{T_{h}D}\left[\bar{D}\right]+{\bar{\lambda }}_{T_{h}M}\left[\bar{M}\right]+{\bar{\lambda }}_{T_{h}\mu_{1}}\left[{\bar{\mu }}_{1}\right]\right)\left[{\bar{T}}_{N}\right]-\left({\bar{\delta }}_{T_{h}\mu_{2}}\left[{\bar{\mu }}_{2}\right]+{\bar{\delta }}_{T_{h}T_{r}}\left[{\bar{T}}_{r}\right]+\delta_{T_{h}}\right)\left[{\bar{T}}_{h}\right] \end{array}$$

(A33) $$\begin{array}{cc} \frac{d\left[{\bar{T}}_{C}\right]}{dt}= & \left({\bar{\lambda }}_{T_{C}T_{h}}\left[{\bar{T}}_{h}\right]+{\bar{\lambda }}_{T_{C}D}\left[\bar{D}\right]\right)\left[{\bar{T}}_{N}\right]-\left({\bar{\delta }}_{T_{C}\mu_{2}}\left[{\bar{\mu }}_{2}\right]+{\bar{\delta }}_{T_{C}T_{r}}\left[{\bar{T}}_{r}\right]+\delta_{T_{C}}\right)\left[{\bar{T}}_{C}\right] \end{array}$$

(A34) $$\begin{array}{cc} \frac{d\left[{\bar{T}}_{r}\right]}{dt}= & \left({\bar{\lambda }}_{T_{r}T_{h}}\left[{\bar{T}}_{h}\right]+{\bar{\lambda }}_{T_{r}\mu_{2}}\left[{\bar{\mu }}_{2}\right]+{\bar{\lambda }}_{T_{r}G_{\beta }}\left[{\bar{G}}_{\beta }\right]\right)\left[{\bar{T}}_{N}\right]-\left({\bar{\delta }}_{T_{r}\mu_{1}}\left[{\bar{\mu }}_{1}\right]+\delta_{T_{r}}\right)\left[{\bar{T}}_{r}\right] \end{array}$$

(A35) $$\begin{array}{cc} \frac{d\left[{\bar{D}}_{N}\right]}{dt}= & {\bar{A}}_{D_{N}}-\alpha_{D_{N}D}\left({\bar{\lambda }}_{DH}\left[\bar{H}\right]+{\bar{\lambda }}_{DC}\left[\bar{C}\right]\right)\left[{\bar{D}}_{N}\right]-\left({\bar{\delta }}_{DH}\left[\bar{H}\right]+\delta_{D}\right)\left[{\bar{D}}_{N}\right] \end{array}$$

(A36) $$\begin{array}{cc} \frac{d\left[\bar{D}\right]}{dt}= & \left({\bar{\lambda }}_{DH}\left[\bar{H}\right]+{\bar{\lambda }}_{DC}\left[\bar{C}\right]\right)\left[{\bar{D}}_{N}\right]-\left({\bar{\delta }}_{DH}\left[\bar{H}\right]+{\bar{\delta }}_{DC}\left[\bar{C}\right]+\delta_{D}\right)\left[\bar{D}\right] \end{array}$$

(A37) $$\begin{array}{cc} \frac{d\left[\bar{M}\right]}{dt}= & \left({\bar{\lambda }}_{M\mu_{2}}\left[{\bar{\mu }}_{2}\right]+{\bar{\lambda }}_{MI_{\gamma }}\left[{\bar{I}}_{\gamma }\right]+{\bar{\lambda }}_{MT_{h}}\left[{\bar{T}}_{h}\right]\right)\left({\bar{M}}_{0}-\left[\bar{M}\right]\right)-\delta_{M}\left[\bar{M}\right] \end{array}$$

(A38) $$\begin{array}{cc} \frac{d\left[\bar{C}\right]}{dt}= & \left(\lambda_{C}+{\bar{\lambda }}_{C\mu_{1}}\left[{\bar{\mu }}_{1}\right]\right)\left[\bar{C}\right]\left(1-\frac{\left[\bar{C}\right]}{{\bar{C}}_{0}}\right)-\left({\bar{\delta }}_{CG_{\beta }}\left[{\bar{G}}_{\beta }\right]+{\bar{\delta }}_{CI_{\gamma }}\left[{\bar{I}}_{\gamma }\right]+{\bar{\delta }}_{CT_{C}}\left[{\bar{T}}_{C}\right]+\delta_{C}\right)\left[\bar{C}\right] \end{array}$$

(A39) $$\begin{array}{cc} \frac{d\left[\bar{N}\right]}{dt}= & {\bar{\alpha }}_{NC}\left({\bar{\delta }}_{CG_{\beta }}\left[{\bar{G}}_{\beta }\right]+{\bar{\delta }}_{CI_{\gamma }}\left[{\bar{I}}_{\gamma }\right]+{\bar{\delta }}_{CT_{C}}\left[{\bar{T}}_{C}\right]+\delta_{C}\right)\left[\bar{C}\right]-\delta_{N}\left[\bar{N}\right] \end{array}$$

(A40) $$\begin{array}{cc} \frac{d\left[\bar{H}\right]}{dt}= & {\bar{\lambda }}_{HN}\left[\bar{N}\right]+{\bar{\lambda }}_{HM}\left[\bar{M}\right]+{\bar{\lambda }}_{HT_{h}}\left[{\bar{T}}_{h}\right]+{\bar{\lambda }}_{HT_{C}}\left[{\bar{T}}_{C}\right]+{\bar{\lambda }}_{HT_{r}}\left[{\bar{T}}_{r}\right]-\delta_{H}\left[\bar{H}\right] \end{array}$$

(A41) $$\begin{array}{cc} \frac{d\left[{\bar{\mu }}_{1}\right]}{dt}= & {\bar{\lambda }}_{\mu_{1}T_{h}}\left[{\bar{T}}_{h}\right]+{\bar{\lambda }}_{\mu_{1}M}\left[\bar{M}\right]+{\bar{\lambda }}_{\mu_{1}D}\left[\bar{D}\right]-\delta_{\mu_{1}}\left[{\bar{\mu }}_{1}\right] \end{array}$$

(A42) $$\begin{array}{cc} \frac{d\left[{\bar{\mu }}_{2}\right]}{dt}= & {\bar{\lambda }}_{\mu_{2}M}\left[\bar{M}\right]+{\bar{\lambda }}_{\mu_{2}D}\left[\bar{D}\right]+{\bar{\lambda }}_{\mu_{2}T_{r}}\left[{\bar{T}}_{r}\right]-\delta_{\mu_{2}}\left[{\bar{\mu }}_{2}\right] \end{array}$$

(A43) $$\begin{array}{cc} \frac{d\left[{\bar{I}}_{\gamma }\right]}{dt}= & {\bar{\lambda }}_{I_{\gamma }T_{h}}\left[{\bar{T}}_{h}\right]+{\bar{\lambda }}_{I_{\gamma }T_{C}}\left[{\bar{T}}_{C}\right]+{\bar{\lambda }}_{I_{\gamma }M}\left[\bar{M}\right]-\delta_{I_{\gamma }}\left[{\bar{I}}_{\gamma }\right] \end{array}$$

(A44) $$\begin{array}{cc} \frac{d\left[{\bar{G}}_{\beta }\right]}{dt}= & {\bar{\lambda }}_{G_{\beta }M}\left[\bar{M}\right]+{\bar{\lambda }}_{G_{\beta }T_{r}}\left[{\bar{T}}_{r}\right]-\delta_{G_{\beta }}\left[{\bar{G}}_{\beta }\right] \end{array}$$

where the nondimensional parameters can be expressed as follows:

$$\begin{array}{cccccccc} {\bar{A}}_{T_{N}}= & \frac{A_{T_{N}}}{T_{N}^{\infty }}, & \alpha_{T_{N}T_{h}}= & \frac{T_{h}^{\infty }}{T_{N}^{\infty }}, & \alpha_{T_{N}T_{C}}= & \frac{T_{C}^{\infty }}{T_{N}^{\infty }}, & \alpha_{T_{N}T_{r}}= & \frac{T_{r}^{\infty }}{T_{N}^{\infty }},\\ {\bar{A}}_{D_{N}}= & \frac{A_{D_{N}}}{D_{N}^{\infty }}, & \alpha_{D_{N}D}= & \frac{D^{\infty }}{D_{N}^{\infty }}, & {\bar{M}}_{0}= & \frac{M_{0}}{M^{\infty }}, & {\bar{C}}_{0}= & \frac{C_{0}}{C^{\infty }},\\ {\bar{\alpha }}_{NC}= & \alpha_{NC}\frac{C^{\infty }}{N^{\infty }}, & {\bar{\lambda }}_{T_{h}D}= & \frac{\lambda_{T_{h}D}D^{\infty }T_{N}^{\infty }}{T_{h}^{\infty }}, & {\bar{\lambda }}_{T_{h}M}= & \frac{\lambda_{T_{h}M}M^{\infty }T_{N}^{\infty }}{T_{h}^{\infty }}, & {\bar{\lambda }}_{T_{h}\mu_{1}}= & \frac{\lambda_{T_{h}\mu_{1}}\mu_{1}^{\infty }T_{N}^{\infty }}{T_{h}^{\infty }},\\ {\bar{\lambda }}_{T_{C}T_{h}}= & \frac{\lambda_{T_{C}T_{h}}T_{h}^{\infty }T_{N}^{\infty }}{T_{C}^{\infty }}, & {\bar{\lambda }}_{T_{C}D}= & \frac{\lambda_{T_{C}D}D^{\infty }T_{N}^{\infty }}{T_{C}^{\infty }}, & {\bar{\lambda }}_{T_{r}T_{h}}= & \frac{\lambda_{T_{r}T_{h}}T_{h}^{\infty }T_{N}^{\infty }}{T_{r}^{\infty }}, & {\bar{\lambda }}_{T_{r}\mu_{2}}= & \frac{\lambda_{T_{r}\mu_{2}}\mu_{2}^{\infty }T_{N}^{\infty }}{T_{r}^{\infty }},\\ {\bar{\lambda }}_{T_{r}G_{\beta }}= & \frac{\lambda_{T_{r}G_{\beta }}G_{\beta }^{\infty }T_{N}^{\infty }}{T_{r}^{\infty }}, & {\bar{\lambda }}_{DH}= & \frac{\lambda_{DH}H^{\infty }D_{N}^{\infty }}{D^{\infty }}, & {\bar{\lambda }}_{DC}= & \frac{\lambda_{DH}C^{\infty }D_{N}^{\infty }}{D^{\infty }}, & {\bar{\lambda }}_{M\mu_{2}}= & \lambda_{M\mu_{2}}\mu_{2}^{\infty },\\ {\bar{\lambda }}_{MI_{\gamma }}= & \lambda_{MI_{\gamma }}I_{\gamma }^{\infty }, & {\bar{\lambda }}_{MT_{h}}= & \lambda_{MT_{h}}T_{h}^{\infty }, & {\bar{\lambda }}_{C\mu_{1}}= & \lambda_{C\mu_{1}}\mu_{1}^{\infty }, & {\bar{\lambda }}_{HN}= & \frac{\lambda_{HN}N^{\infty }}{H^{\infty }},\\ {\bar{\lambda }}_{HM}= & \frac{\lambda_{HM}M^{\infty }}{H^{\infty }}, & {\bar{\lambda }}_{HT_{h}}= & \frac{\lambda_{HT_{h}}T_{h}^{\infty }}{H^{\infty }}, & {\bar{\lambda }}_{HT_{C}}= & \frac{\lambda_{HT_{C}}T_{C}^{\infty }}{H^{\infty }}, & {\bar{\lambda }}_{HT_{r}}= & \frac{\lambda_{HT_{r}}T_{r}^{\infty }}{H^{\infty }},\\ {\bar{\lambda }}_{\mu_{1}T_{h}}= & \frac{\lambda_{\mu_{1}T_{h}}T_{h}^{\infty }}{\mu_{1}^{\infty }}, & {\bar{\lambda }}_{\mu_{1}M}= & \frac{\lambda_{\mu_{1}M}M^{\infty }}{\mu_{1}^{\infty }}, & {\bar{\lambda }}_{\mu_{1}D}= & \frac{\lambda_{\mu_{1}D}D^{\infty }}{\mu_{1}^{\infty }}, & {\bar{\lambda }}_{\mu_{2}M}= & \frac{\lambda_{\mu_{2}M}M^{\infty }}{\mu_{2}^{\infty }},\\ {\bar{\lambda }}_{\mu_{2}D}= & \frac{\lambda_{\mu_{2}D}D^{\infty }}{\mu_{2}^{\infty }}, & {\bar{\lambda }}_{\mu_{2}T_{r}}= & \frac{\lambda_{\mu_{2}T_{r}}T_{r}^{\infty }}{\mu_{2}^{\infty }}, & {\bar{\lambda }}_{I_{\gamma }T_{h}}= & \frac{\lambda_{I_{\gamma }T_{h}}T_{h}^{\infty }}{I_{\gamma }^{\infty }}, & {\bar{\lambda }}_{I_{\gamma }T_{C}}= & \frac{\lambda_{I_{\gamma }T_{C}}T_{C}^{\infty }}{I_{\gamma }^{\infty }},\\ {\bar{\lambda }}_{I_{\gamma }M}= & \frac{\lambda_{I_{\gamma }M}M^{\infty }}{I_{\gamma }^{\infty }}, & {\bar{\lambda }}_{G_{\beta }M}= & \frac{\lambda_{G_{\beta }M}M^{\infty }}{G_{\beta }^{\infty }}, & {\bar{\lambda }}_{G_{\beta }T_{r}}= & \frac{\lambda_{G_{\beta }T_{r}}T_{r}^{\infty }}{G_{\beta }^{\infty }}, & {\bar{\delta }}_{T_{h}\mu_{2}}= & \delta_{T_{h}\mu_{2}}\mu_{2}^{\infty },\\ {\bar{\delta }}_{T_{h}T_{r}}= & \delta_{T_{h}T_{r}}T_{r}^{\infty }, & {\bar{\delta }}_{T_{C}\mu_{2}}= & \delta_{T_{C}\mu_{2}}\mu_{2}^{\infty }, & {\bar{\delta }}_{T_{C}T_{r}}= & \delta_{T_{C}T_{r}}T_{r}^{\infty }, & {\bar{\delta }}_{T_{r}\mu_{1}}= & \delta_{T_{r}\mu_{1}}\mu_{1}^{\infty },\\ {\bar{\delta }}_{DH}= & \delta_{DH}H^{\infty }, & {\bar{\delta }}_{DC}= & \delta_{DC}C^{\infty }, & {\bar{\delta }}_{CG_{\beta }}= & \delta_{CG_{\beta }}G_{\beta }^{\infty }, & {\bar{\delta }}_{CI_{\gamma }}= & \delta_{CI_{\gamma }}I_{\gamma }^{\infty },\\ {\bar{\delta }}_{CT_{C}}= & \delta_{CT_{C}}T_{C}^{\infty }. \end{array}$$

Cancer proliferation rate $\lambda_{C}$ and all the innate degradation/death rates remain unscaled.

Then the equations for doubling rate (A18) and (A19) become

$$\begin{array}{cc} \left(\lambda_{C}+{\bar{\lambda }}_{T_{h}\mu_{1}}\frac{\mu_{1}^{\mathrm{mean}}}{\mu_{1}^{\infty }}\right)-\delta_{C} & =7.5\times 10^{-3},\\ \lambda_{C}-\left({\bar{\delta }}_{CG_{\beta }}\frac{G_{\beta }^{\mathrm{mean}}}{G_{\beta }^{\infty }}+{\bar{\delta }}_{CI_{\gamma }}\frac{I_{\gamma }^{\mathrm{mean}}}{I_{\gamma }^{\infty }}+{\bar{\delta }}_{CT_{C}}\frac{T_{C}^{\mathrm{mean}}}{T_{C}^{\infty }}+\delta_{C}\right) & =6.7152\times 10^{-4}, \end{array}$$

and the system of restrictions (A20)–(A30) in dimensionless form can be rewritten as

$$\begin{array}{cccc} {\bar{\lambda }}_{T_{h}D}\frac{D^{\max}}{D^{\infty }}= & 200{\bar{\lambda }}_{T_{h}M}\frac{M^{\max}}{M^{\infty }}=200{\bar{\lambda }}_{T_{h}\mu_{1}}\frac{\mu_{1}^{\max}}{\mu_{1}^{\infty }},\; & {\bar{\delta }}_{T_{h}\mu_{2}}\frac{\mu_{2}^{\max}}{\mu_{2}^{\infty }}= & {\bar{\delta }}_{T_{h}T_{r}}\frac{T_{r}^{\max}}{T_{r}^{\infty }}=20{\bar{\delta }}_{T_{h}},\;\\ {\bar{\lambda }}_{T_{C}T_{h}}\frac{T_{h}^{\max}}{T_{h}^{\infty }}= & 2{\bar{\lambda }}_{T_{C}D}\frac{D^{\max}}{D^{\infty }}, & {\bar{\delta }}_{T_{C}\mu_{2}}\frac{\mu_{2}^{\max}}{\mu_{2}^{\infty }}= & {\bar{\delta }}_{T_{C}T_{r}}\frac{T_{r}^{\max}}{T_{r}^{\infty }}=20{\bar{\delta }}_{T_{C}},\;\\ {\bar{\lambda }}_{T_{r}T_{h}}\frac{T_{h}^{\max}}{T_{h}^{\infty }}= & 4{\bar{\lambda }}_{T_{r}G_{\beta }}\frac{G_{\beta }^{\max}}{G_{\beta }^{\infty }},\; & {\bar{\lambda }}_{T_{r}\mu_{2}}\frac{\mu_{2}^{\max}}{\mu_{2}^{\infty }}= & {\bar{\lambda }}_{T_{r}G_{\beta }}\frac{G_{\beta }^{\max}}{G_{\beta }^{\infty }},\\ {\bar{\delta }}_{T_{r}\mu_{1}}\frac{\mu_{1}^{\max}}{\mu_{1}^{\infty }}= & 20{\bar{\delta }}_{T_{r}},\; & {\bar{\lambda }}_{DH}\frac{H^{\max}}{H^{\infty }}= & 2{\bar{\lambda }}_{DC}\frac{C^{\max}}{C^{\infty }},\;\\ {\bar{\delta }}_{DH}\frac{H^{\max}}{H^{\infty }}= & \frac{1}{2}{\bar{\delta }}_{DC}\frac{C^{\max}}{C^{\infty }}=\frac{1}{3}{\bar{\delta }}_{D}, & {\bar{\lambda }}_{MT_{h}}\frac{T_{h}^{\max}}{T_{h}^{\infty }}= & 10{\bar{\lambda }}_{M\mu_{2}}\frac{\mu_{2}^{\max}}{\mu_{2}^{\infty }}=10{\bar{\lambda }}_{MI_{\gamma }}\frac{I_{\gamma }^{\max}}{I_{\gamma }^{\infty }},\;\\ {\bar{\delta }}_{CG_{\beta }}\frac{G_{\beta }^{\max}}{G_{\beta }^{\infty }}= & 10\delta_{C} & {\bar{\delta }}_{CG_{\beta }}\frac{G_{\beta }^{\max}}{G_{\beta }^{\infty }}= & {\bar{\delta }}_{CI_{\gamma }}\frac{I_{\gamma }^{\max}}{I_{\gamma }^{\infty }}=\frac{1}{2}{\bar{\delta }}_{CT_{C}}\frac{T_{C}^{\max}}{T_{C}^{\infty }},\\ \frac{{\bar{\lambda }}_{\mu_{1}M}}{M^{\infty }}= & 4\frac{{\bar{\lambda }}_{\mu_{1}T_{h}}}{T_{h}^{\infty }}=8\frac{{\bar{\lambda }}_{\mu_{1}D}}{D^{\infty }},\; & \frac{{\bar{\lambda }}_{\mu_{2}D}}{D^{\infty }}= & \frac{{\bar{\lambda }}_{\mu_{2}M}}{M^{\infty }}=\frac{{\bar{\lambda }}_{\mu_{2}T_{r}}}{T_{r}^{\infty }},\;\\ \frac{{\bar{\lambda }}_{HN}}{N^{\infty }}= & 10\frac{{\bar{\lambda }}_{HM}}{M^{\infty }}=20\frac{{\bar{\lambda }}_{HT_{h}}}{T_{h}^{\infty }}, & \frac{{\bar{\lambda }}_{HT_{h}}}{T_{h}^{\infty }}= & \frac{{\bar{\lambda }}_{HT_{C}}}{T_{C}^{\infty }}=\frac{{\bar{\lambda }}_{HT_{r}}}{T_{r}^{\infty }},\;\\ \frac{{\bar{\lambda }}_{I_{\gamma }T_{C}}}{T_{C}^{\infty }}= & 4\frac{{\bar{\lambda }}_{I_{\gamma }T_{h}}}{T_{h}^{\infty }}=20\frac{{\bar{\lambda }}_{I_{\gamma }M}}{M^{\infty }},\; & \frac{{\bar{\lambda }}_{G_{\beta }M}}{M^{\infty }}= & \frac{{\bar{\lambda }}_{G_{\beta }T_{r}}}{T_{r}^{\infty }}. \end{array}$$

These 29 restriction, together with 14 equations from requiring the steady-state of (A31)–(A44), and 11 given decay rates (A15)–(A17), scaling coefficients

$$\begin{array}{cccccccccc} \alpha_{T_{N}T_{h}}= & \frac{T_{h}^{\infty }}{T_{N}^{\infty }}, & \alpha_{T_{N}T_{C}}= & \frac{T_{C}^{\infty }}{T_{N}^{\infty }}, & \alpha_{T_{N}T_{r}}= & \frac{T_{r}^{\infty }}{T_{N}^{\infty }}, & \alpha_{D_{N}D}= & \frac{D^{\infty }}{D_{N}^{\infty }}, & {\bar{\alpha }}_{NC}= & \alpha_{NC}\frac{C^{\infty }}{N^{\infty }}, \end{array}$$

and given $\alpha_{NC}$, ${\bar{C}}_{0}$ and ${\bar{M}}_{0}$ are enough to derive all 63 non-dimensional coefficients of (A31)–(A44) from

$$T_{N}^{\infty },\;T_{h}^{\infty },\;T_{C}^{\infty },\;T_{r}^{\infty },\;D_{N}^{\infty },\;D^{\infty },\;M^{\infty },\;C^{\infty },\;N^{\infty },\;H^{\infty },\;\mu_{1}^{\infty },\;\mu_{2}^{\infty },\;I_{\gamma }^{\infty },\;G_{\beta }^{\infty }.$$

### Appendix B.3. Parameter Values

Here we detail all the parameter values derived and used in this paper. We divide them into three groups: innate degradation rates derived or adopted from prior research (see Table A1), scaling-dependent parameters (see Table A2) and scaling-independent parameters (those not affected by non-dimensionalization, see Table A3). It should be emphasized that for analyzing the dynamics of the tumor based on the estimated parameters, we should consider possible variations in the assumptions of Appendix B.1 as well as variations between patients. We have tried to take into account these variations by performing sensitivity analysis and clustering patients based on their immune variations.

The scaling-independent parameters parameters include innate cancer proliferation rate $\lambda_{C}$, innate cancer death rate (including natural death and necrosis) $\delta_{C}$ and necrotic cell degradation rate $\delta_{N}$. As a result that these parameters were not affected by non-dimensionalization procedure, as they are determined they remain independent of the scaling constant $\alpha_{dim}$, and depend solely on the derivation assumptions and patient data (thus different between clusters).

The scaling dependent parameters in their dimensional form in addition to derivation assumptions and patient data would also depend on the scaling constant $\alpha_{dim}$. Thus, we prefer to list their more objective non-dimensional values. As a result that non-dimensionalization was done without time scaling, the dimension of most of these parameters is day^−1^. Exceptions are ${\bar{\alpha }}_{NC}$, ${\bar{M}}_{0}$, ${\bar{C}}_{0}$, $\alpha_{T_{N}T_{h}}$, $\alpha_{T_{N}T_{C}}$, $\alpha_{T_{N}T_{r}}$, $\alpha_{D_{N}D}$, these are fully non-dimensional.

**Table A1:** **Prescribed parameters and their references.** Innate degradation and death rates (in day^−1^) derived or adopted from given references.

Parameter	$\delta_{T_{N}}$	$\delta_{T_{h}}$	$\delta_{T_{C}}$	$\delta_{T_{r}}$	$\delta_{D}$	$\delta_{M}$	$\delta_{\mu_{1}}$	$\delta_{\mu_{2}}$	$\delta_{H}$	$\delta_{I_{\gamma }}$	$\delta_{G_{\beta }}$
Value	9.4951 $\times \;10^{-4}$	0.231	0.406	0.231	0.277	0.02	1.07	4.62	58.7	33.27	499.0
Reference	[154]	[150]	[150]	[150]	[149]	[151,158]	[153]	[155]	[152]	[157]	[156]

**Table A2:** **Scaling-dependent parameters.** Non-dimensional values of scaling-dependent parameters (in day^−1^, because the time was not scaled) for each cluster derived from the steady-state assumptions and patient data.

Parameter	Cluster 1	Cluster 2	Cluster 3	Cluster 4	Cluster 5
${\bar{\lambda }}_{T_{h}D}$	2.1399	2.7619	2.3163	1.7613	2.0179
${\bar{\lambda }}_{T_{h}M}$	3.9171 $\times \;10^{-2}$	4.3371 $\times \;10^{-2}$	7.8933 $\times \;10^{-2}$	3.4235 $\times \;10^{-2}$	1.1462 $\times \;10^{-1}$
${\bar{\lambda }}_{T_{h}\mu_{1}}$	1.1871 $\times \;10^{-2}$	1.9332 $\times \;10^{-2}$	5.0516 $\times \;10^{-2}$	6.6146 $\times \;10^{-3}$	2.6083 $\times \;10^{-2}$
${\bar{\lambda }}_{T_{C}T_{h}}$	3.2503	4.4295	3.6087	2.3377	3.5400
${\bar{\lambda }}_{T_{C}D}$	6.0053 $\times \;10^{-1}$	5.3505 $\times \;10^{-1}$	6.8998 $\times \;10^{-1}$	8.2970 $\times \;10^{-1}$	2.5392 $\times \;10^{-1}$
${\bar{\lambda }}_{T_{r}T_{h}}$	8.2898 $\times \;10^{-1}$	8.9260 $\times \;10^{-1}$	7.1048 $\times \;10^{-1}$	6.6821 $\times \;10^{-1}$	6.4634 $\times \;10^{-1}$
${\bar{\lambda }}_{T_{r}\mu_{2}}$	5.1120 $\times \;10^{-2}$	4.3090 $\times \;10^{-2}$	1.4558 $\times \;10^{-1}$	1.9891 $\times \;10^{-2}$	2.6898 $\times \;10^{-2}$
${\bar{\lambda }}_{T_{r}G_{\beta }}$	6.4120 $\times \;10^{-2}$	6.1990 $\times \;10^{-2}$	2.4271 $\times \;10^{-1}$	4.1296 $\times \;10^{-2}$	8.4474 $\times \;10^{-2}$
${\bar{\lambda }}_{DH}$	2.6134 $\times \;10^{-1}$	2.5410 $\times \;10^{-1}$	2.6749 $\times \;10^{-1}$	2.0531 $\times \;10^{-1}$	2.5253 $\times \;10^{-1}$
${\bar{\lambda }}_{DC}$	1.0129 $\times \;10^{-1}$	1.0998 $\times \;10^{-1}$	9.6275 $\times \;10^{-2}$	1.4619 $\times \;10^{-1}$	9.9406 $\times \;10^{-2}$
${\bar{\lambda }}_{M\mu_{2}}$	6.0389 $\times \;10^{-4}$	4.0918 $\times \;10^{-4}$	4.0357 $\times \;10^{-4}$	1.0688 $\times \;10^{-3}$	2.2488 $\times \;10^{-4}$
${\bar{\lambda }}_{MI_{\gamma }}$	5.4536 $\times \;10^{-4}$	4.4647 $\times \;10^{-4}$	3.5449 $\times \;10^{-5}$	5.5129 $\times \;10^{-4}$	2.4891 $\times \;10^{-4}$
${\bar{\lambda }}_{MT_{h}}$	2.4482 $\times \;10^{-2}$	2.1190 $\times \;10^{-2}$	4.9239 $\times \;10^{-3}$	8.9758 $\times \;10^{-2}$	1.3509 $\times \;10^{-2}$
${\bar{\lambda }}_{C\mu_{1}}$	3.6453 $\times \;10^{-3}$	5.6528 $\times \;10^{-3}$	1.3143 $\times \;10^{-3}$	2.5321 $\times \;10^{-3}$	2.5536 $\times \;10^{-3}$
${\bar{\alpha }}_{NC}$	1.5	1.5	1.5	1.5	1.5
${\bar{\lambda }}_{\mu_{1}T_{h}}$	9.5154 $\times \;10^{-2}$	1.5848 $\times \;10^{-1}$	5.1746 $\times \;10^{-2}$	4.8884 $\times \;10^{-2}$	8.0520 $\times \;10^{-2}$
${\bar{\lambda }}_{\mu_{1}M}$	9.6867 $\times \;10^{-1}$	9.0479 $\times \;10^{-1}$	1.0148	1.0150	9.8745 $\times \;10^{-1}$
${\bar{\lambda }}_{\mu_{1}D}$	6.1798 $\times \;10^{-3}$	6.7287 $\times \;10^{-3}$	3.4777 $\times \;10^{-3}$	6.0984 $\times \;10^{-3}$	2.0302 $\times \;10^{-3}$
${\bar{\lambda }}_{\mu_{2}M}$	3.6856	3.1869	3.2127	3.7737	3.7140
${\bar{\lambda }}_{\mu_{2}D}$	1.8810 $\times \;10^{-1}$	1.8960 $\times \;10^{-1}$	8.8081 $\times \;10^{-2}$	1.8139 $\times \;10^{-1}$	6.1088 $\times \;10^{-2}$
${\bar{\lambda }}_{\mu_{2}T_{r}}$	7.4634 $\times \;10^{-1}$	1.2435	1.3192	6.6490 $\times \;10^{-1}$	8.4487 $\times \;10^{-1}$
${\bar{\lambda }}_{HN}$	5.6645 ×10	5.6824 ×10	5.7494 ×10	5.6720 ×10	5.6504 ×10
${\bar{\lambda }}_{HM}$	1.4603	1.0096	8.6817 $\times \;10^{-1}$	1.5130	1.6271
${\bar{\lambda }}_{HT_{h}}$	2.8689 $\times \;10^{-1}$	3.5366 $\times \;10^{-1}$	8.8539 $\times \;10^{-2}$	1.4573 $\times \;10^{-1}$	2.6536 $\times \;10^{-1}$
${\bar{\lambda }}_{HT_{C}}$	1.5995 $\times \;10^{-1}$	3.1527 $\times \;10^{-1}$	7.1133 $\times \;10^{-2}$	1.8821 $\times \;10^{-1}$	1.1817 $\times \;10^{-1}$
${\bar{\lambda }}_{HT_{r}}$	1.4786 $\times \;10^{-1}$	1.9697 $\times \;10^{-1}$	1.7824 $\times \;10^{-1}$	1.3328 $\times \;10^{-1}$	1.8507 $\times \;10^{-1}$
${\bar{\lambda }}_{I_{\gamma }T_{h}}$	8.8980	6.8580	6.4053	4.6181	9.8011
${\bar{\lambda }}_{I_{\gamma }T_{C}}$	1.9843 ×10	2.4454 ×10	2.0584 ×10	2.3857 ×10	1.7459 ×10
${\bar{\lambda }}_{I_{\gamma }M}$	4.5290	1.9577	6.2806	4.7945	6.0098
${\bar{\lambda }}_{G_{\beta }M}$	4.1497 $\times \;10^{2}$	3.5894 $\times \;10^{2}$	3.5375 $\times \;10^{2}$	4.2425 $\times \;10^{2}$	4.0652 $\times \;10^{2}$
${\bar{\lambda }}_{G_{\beta }T_{r}}$	8.4033 ×10	1.4006 $\times \;10^{2}$	1.4525 $\times \;10^{2}$	7.4749 ×10	9.2476 ×10
${\bar{\delta }}_{T_{h}\mu_{2}}$	4.2911 $\times \;10^{-1}$	4.3775 $\times \;10^{-1}$	4.2642 $\times \;10^{-1}$	1.1131 $\times \;10^{-1}$	2.3641 $\times \;10^{-1}$
${\bar{\delta }}_{T_{h}T_{r}}$	1.5309	2.1559	1.7884	1.4599	1.6912
${\bar{\delta }}_{T_{C}\mu_{2}}$	7.5419 $\times \;10^{-1}$	7.6938 $\times \;10^{-1}$	7.4947 $\times \;10^{-1}$	1.9563 $\times \;10^{-1}$	4.1550 $\times \;10^{-1}$
${\bar{\delta }}_{T_{C}T_{r}}$	2.6906	3.7891	3.1432	2.5658	2.9724
${\bar{\delta }}_{T_{r}\mu_{1}}$	7.1322 $\times \;10^{-1}$	7.6668 $\times \;10^{-1}$	8.6777 $\times \;10^{-1}$	4.9839 $\times \;10^{-1}$	5.2671 $\times \;10^{-1}$
${\bar{\delta }}_{DH}$	3.3577 $\times \;10^{-2}$	3.1883 $\times \;10^{-2}$	3.5563 $\times \;10^{-2}$	1.9360 $\times \;10^{-2}$	2.9105 $\times \;10^{-2}$
${\bar{\delta }}_{DC}$	5.2053 $\times \;10^{-2}$	5.5200 $\times \;10^{-2}$	5.1199 $\times \;10^{-2}$	5.5139 $\times \;10^{-2}$	4.5828 $\times \;10^{-2}$
${\bar{\delta }}_{CG_{\beta }}$	9.1600 $\times \;10^{-4}$	7.1011 $\times \;10^{-4}$	1.8590 $\times \;10^{-3}$	3.9506 $\times \;10^{-4}$	1.3131 $\times \;10^{-3}$
${\bar{\delta }}_{CI_{\gamma }}$	6.5951 $\times \;10^{-4}$	5.3859 $\times \;10^{-4}$	9.7944 $\times \;10^{-5}$	9.8156 $\times \;10^{-5}$	4.6278 $\times \;10^{-4}$
${\bar{\delta }}_{CT_{C}}$	2.1270 $\times \;10^{-3}$	2.9365 $\times \;10^{-3}$	1.4085 $\times \;10^{-3}$	2.6597 $\times \;10^{-3}$	1.4414 $\times \;10^{-3}$
${\bar{A}}_{T_{N}}$	1.5006	4.1268	1.2183	1.1785	1.6150
${\bar{A}}_{Dn}$	1.0264	2.1172	3.5941 $\times \;10^{2}$	1.8353	1.1435
${\bar{M}}_{0}$	1.7803	1.9072	4.7293	1.2189	2.4303
${\bar{C}}_{0}$	2.0	2.0	2.0	2.0	2.0
$\alpha_{T_{N}T_{h}}$	3.1084 $\times \;10^{-1}$	5.2856 $\times \;10^{-1}$	1.5008 $\times \;10^{-1}$	1.7950 $\times \;10^{-1}$	3.6879 $\times \;10^{-1}$
$\alpha_{T_{N}T_{C}}$	1.7330 $\times \;10^{-1}$	4.7118 $\times \;10^{-1}$	1.2057 $\times \;10^{-1}$	2.3182 $\times \;10^{-1}$	1.6423 $\times \;10^{-1}$
$\alpha_{T_{N}T_{r}}$	1.6020 $\times \;10^{-1}$	2.9438 $\times \;10^{-1}$	3.0212 $\times \;10^{-1}$	1.6417 $\times \;10^{-1}$	2.5720 $\times \;10^{-1}$
$\alpha_{D_{N}D}$	1.9739	4.9667	9.8717 $\times \;10^{2}$	4.3783	2.3796

**Table A3:** **Scaling-independent parameters.** Values of scaling-independent parameters (in day${}^{-1}$) for each cluster derived from the steady-state assumptions and patient data.

Parameter	Cluster 1	Cluster 2	Cluster 3	Cluster 4	Cluster 5
$\lambda_{C}$	5.3323 $\times \;10^{-3}$	3.7596 $\times \;10^{-3}$	7.8327 $\times \;10^{-3}$	5.3535 $\times \;10^{-3}$	5.5150 $\times \;10^{-3}$
$\delta_{C}$	7.8626 $\times \;10^{-4}$	5.2094 $\times \;10^{-4}$	1.2081 $\times \;10^{-3}$	7.8983 $\times \;10^{-4}$	8.1708 $\times \;10^{-4}$
$\delta_{N}$	6.7332 $\times \;10^{-3}$	7.0593 $\times \;10^{-3}$	6.8602 $\times \;10^{-3}$	5.9142 $\times \;10^{-3}$	6.0514 $\times \;10^{-3}$

## Appendix C. Patient Data and Initial Conditions

Here we describe the processing of the data to be used for parameter estimation and initial conditions. The clustered deconvolution data, described in Section 2.3, and original TCGA data are used to calculate the immune variables as described in Table A4.

**Table A4:** **Patient data correspondence with variables.** Correspondence between the system variables and the source data from TCGA and deconvolution results.

Variable	Data Used
$T_{N}$	T cells CD4 naive, T cells CD4 memory resting, NK cells resting
$T_{h}$	T cells CD4 memory activated, T cells follicular helper
$T_{C}$	T cells CD8, NK cells activated
$T_{r}$	T cells regulatory (Tregs)
$D_{N}$	Dendritic cells resting
*D*	Dendritic cells activated
*M*	Macrophages M1, Macrophages M2
$M_{0}$	Monocytes, Macrophages M0, Macrophages M1, Macrophages M2
$\mu_{1}$	IL6, IL17A, IL17B, IL17C, IL17D, IL17F, IL21, IL22
$\mu_{2}$	CCL20, IL10
*H*	HMGB1
$I_{\gamma }$	IFNG
$G_{\beta }$	TGFB1, TGFB1I1, TGFB2, TGFB3, TGFBI
size(P)	multiply_dimensions
Total_Immune_Density	$T_{N}$, $T_{h}$, $T_{C}$, $T_{r}$, $D_{N}$, *D*, $M_{0}$

For variables related to immune cells, we substitute zero values with 10% of the smallest positive cell density for numerical stability.

We estimate the relative amount of cancer cells and necrotic cells as follows: we start by assuming that on average across all patients the ratio of immune cells:cancer cells:necrotic cells will be approximately 0.4:0.4:0.2 with variability between clusters based on tumor size. For patient *P*, we consider tumor size (size(P)) to be the product of the longest dimension and the shortest dimension. We assume total cell density at the steady-state to be proportional to this product as

$$\mathrm{Total}\_\mathrm{Cell}\_{\mathrm{Density}}_{P}=\alpha_{dim}\frac{\mathrm{size}(P)}{\frac{1}{K}\sum_{\mathrm{all}\;P}\mathrm{size}\left(P\right)}.$$

Tumor deconvolution data only provide us with ratios of immune cells relative to each other. Thus, to properly adjust the scaling, we take each immune cell value from deconvolution multiplied by $0.4\alpha_{dim}$, and consider $0.4\alpha_{dim}\sum \left(\mathrm{Immune}\;\mathrm{cell}\;\mathrm{ratios}\right)$ as total immune density and

(A45) $$C=\frac{2}{3}\left(\mathrm{Total}\_\mathrm{Cell}\_\mathrm{Density}-\mathrm{Total}\_\mathrm{Immune}\_\mathrm{Density}\right),\;N=0.5C.$$

Next, for each cluster, we divide patients into three groups according to their their tumor size: above average, below average and no data. Resulting patient numbers of each group are given in Table A5. We use the means from the group “above average” as steady-state assumptions given in Table 2.

**Table A5:** **Distribution of patients according to their tumor size.** Evaluated relative to the average tumor size within each cluster.

	Cluster 1	Cluster 2	Cluster 3	Cluster 4	Cluster 5
Total patients	114	47	29	40	98
Above average	36	15	7	17	36
Below average	48	20	12	23	44
No data	30	12	10	0	18

The data in the “below average” group as evaluated by (A45) may contain negative values for cancer and necrotic cells. The data in “no data” group have no values for cancer and necrotic cells. Thus, we substitute all non-positive and absent cancer values with 10% of the smallest positive cancer density value. We substitute all non-positive and absent necrotic cell values with zero. These changes violate the 0.4:0.4:0.2 ratio of immune cells:cancer cells:necrotic cells, and the updated ratio is 0.4475:0.3684:0.1841.

The steady-state assumptions (see Appendix B) are partially based on maximum values of each variable in the ODE system (A1)–(A14) across all patients, as well as mean value of variables $T_{C},\;\mu_{1},\;I_{\gamma }$ and $G_{\beta }$ across all patients. The corresponding values are given in Table A6.

**Table A6:** **Maximum and mean variable values for parameter estimation.** Maximum and mean cell densities in cells/cm^3^ and cytokine expression levels across all patients used in Appendix B to derive parameter sets for time-dependent solutions.

$T_{N}^{\max}$	$T_{h}^{\max}$	$T_{C}^{\max}$	$T_{r}^{\max}$	$D_{N}^{\max}$	$D^{\max}$
2.6731 $\times \;10^{4}$	1.2311 $\times \;10^{4}$	1.9107 $\times \;10^{4}$	7.2102 $\times \;10^{3}$	3.4173 $\times \;10^{3}$	4.3275 $\times \;10^{3}$
$M^{\max}$	$C^{\max}$	$N^{\max}$	$\mu_{1}^{\max}$	$\mu_{2}^{\max}$	$H^{\max}$
2.3160 $\times \;10^{4}$	3.2472 $\times \;10^{5}$	1.6236 $\times \;10^{5}$	1.0577 $\times \;10^{3}$	1.3983 $\times \;10^{4}$	2.4697 $\times \;10^{4}$
$I_{\gamma }^{\max}$	$G_{\beta }^{\max}$	$T_{C}^{\mathrm{mean}}$	$\mu_{1}^{\mathrm{mean}}$	$I_{\gamma }^{\mathrm{mean}}$	$G_{\beta }^{\mathrm{mean}}$
1.0221 $\times \;10^{2}$	1.6341 $\times \;10^{5}$	2.9203 $\times \;10^{3}$	1.3232 $\times \;10^{2}$	6.6035	2.0018 $\times \;10^{4}$

In each cluster, a patient with the smallest known tumor size is used as initial condition (given in Table 3) for the dynamics computations presented in Figure 5 and Figure 6. However, it is interesting to look at the dynamics of one cluster with initial conditions from another. Figure A1 shows the dynamics of each cluster with every initial condition from all clusters. It demonstrates that the curves originating from different initial conditions quickly converge to the same dynamics, more influenced by the parameters and not the initial conditions. Additionally, any patient in the “below average” group can be reasonably used as initial condition. The resulting dynamics is given by cluster on Figure A2, Figure A3, Figure A4, Figure A5, Figure A6. The colors on the plot correspond to tumor size categories introduced on Figure 3F. These results suggest that the differences in the dynamics within the same cluster are mostly due to the variations in the initial tumor size.
